# Childhood socioeconomic status is associated with psychometric intelligence and microstructural brain development

**DOI:** 10.1038/s42003-021-01974-w

**Published:** 2021-04-29

**Authors:** Hikaru Takeuchi, Yasuyuki Taki, Kohei Asano, Michiko Asano, Yuko Sassa, Susumu Yokota, Yuka Kotozaki, Rui Nouchi, Ryuta Kawashima

**Affiliations:** 1 Division of Developmental Cognitive Neuroscience, Institute of Development, Aging and Cancer, Tohoku University, Sendai, Japan; 2 Division of Medical Neuroimaging Analysis, Department of Community Medical Supports, Tohoku Medical Megabank Organization, Tohoku University, Sendai, Japan; 3 Department of Nuclear Medicine & Radiology, Institute of Development, Aging and Cancer, Tohoku University, Sendai, Japan; 4 Kokoro Research Center, Kyoto University, Kyoto, Japan; 5 Department of Child and Adolescent Mental Health, National Center of Neurology and Psychiatry, Tokyo, Japan; 6 Division for Experimental Natural Science, Faculty of Arts and Science, Kyushu University, Fukuoka, Japan; 7 Division of Clinical research, Medical-Industry Translational Research Center, Fukushima Medical University School of Medicine, Fukushima, Japan; 8 Department of Cognitive Health Science, Institute of Development, Aging and Cancer, Tohoku University, Sendai, Japan; 9 Smart Aging Research Center, Tohoku University, Sendai, Japan; 10 Department of Advanced Brain Science, Institute of Development, Aging and Cancer, Tohoku University, Sendai, Japan

**Keywords:** Intelligence, Human behaviour

## Abstract

Childhood socioeconomic status is robustly associated with various children’s cognitive factors and neural mechanisms. Here we show the association of childhood socioeconomic status with psychometric intelligence and mean diffusivity and fractional anisotropy using diffusion tensor imaging at the baseline experiment (*N* = 285) and longitudinal changes in these metrics after 3.0 ± 0.3 years (*N* = 223) in a large sample of normal Japanese children (mean age = 11.2 ± 3.1 years). After correcting for confounding factors, cross-sectional and longitudinal analyses show that higher childhood socioeconomic status is associated with greater baseline and baseline to follow-up increase of psychometric intelligence and mean diffusivity in areas around the bilateral fusiform gyrus. These results demonstrate that higher socioeconomic status is associated with higher psychometric intelligence measures and altered microstructural properties in the fusiform gyrus which plays a key role in reading and letter recognition and further augmentation of such tendencies during development. Definitive conclusions regarding the causality of these relationships requires intervention and physiological studies. However, the current findings should be considered when developing and revising policies regarding education.

## Abstract (web summary)

Takeuchi et al perform cross-sectional and longitudinal analyses of associations between childhood socioeconomic status and psychometric intelligence as well as microstructural brain development in Japanese children. They report that higher socioeconomic status is associated with greater psychometric intelligence and altered microstructural properties in the fusiform gyrus which plays a role in reading and letter recognition.

## Introduction

Previous work has indicated that childhood socioeconomic status (SES) is robustly associated with a number of cognitive factors and neural mechanisms. In children, it has been documented that higher childhood SES is associated with greater psychometric intelligence, working memory capacity, reading skills, literacy, academic performance^1^. It has been suggested that these associations with working memory are mediated by prefrontal cortical function. In turn, prefrontal cortical function is affected by stress levels, which is linked to individual differences in childhood SES^2,3^.

Previous cross-sectional neuroimaging studies have revealed that childhood SES is associated with brain structures. In young children, childhood SES is associated with quantitative gray matter measures in the prefrontal cortex, medial temporal lobe, and language-related areas^4–6^. Although many studies investigating quantitative gray matter measures have small sample sizes that contribute to inconsistent results, higher or lower childhood SES is generally associated with greater or smaller total and regional quantitative gray matter levels across developmental stages, respectively^4,6–10^. These include associations between low family income and lower hippocampal volume among children aged 4–18 years^4^, associations between low family income and lower frontal, temporal, and hippocampal volume among children to young adults aged 4–22 years^7^, associations between parental education and lower cortical thickness in frontal areas among children aged 4.5–18.3 years^8^, associations between low family income and smaller white and cortical gray matter, hippocampus, and amygdala volumes among children aged 6–12 years^9^, cross-sectional associations between lower family SES and smaller total gray and white matter volume as well as subcortical structures among children to young adults aged 5.2–25.4 years^10^, associations between lower family income and smaller brain surface area among children aged 3–20 years^11^, associations between lower SES and smaller volumes of gray and white matter of the inferior frontal gyrus^12^, associations between lower SES and smaller volumes of cortical and deep gray matter among infants aged 4–6 weeks^13^, and associations between lower childhood SES and smaller volumes of gray matter (frontal and parietal cortices) among infants aged 5 months–4 years^14^. Further, there is a significant relationship between greater childhood SES and greater fractional anisotropy (FA), often interpreted as strength of white matter structural connectivity, in white matter areas adjacent to the hippocampus and corticostriatal white matter tracts in the frontal and parietal cortices among children 3–20 years^15^. Fewer longitudinal studies have been reported, but some recent neuroimaging studies have utilized longitudinal data to investigate the associations of childhood SES with brain development. McDermott et al.^10^ reported that childhood SES was negatively correlated with longitudinal cortical thickness changes in the left middle temporal parietal gyrus and left superior parietal lobule among children and young adults aged 5.2–25.4 years.

However, to date, no study has used a longitudinal design to investigate the associations of childhood SES with changes in microstructural properties in the brain during development as measured by diffusion tensor imaging (DTI). The purpose of this study was to investigate this issue. Utilizing a longitudinal design allows for observation of whether childhood SES, which is defined as the sum of the *z* score of family income and average of parents’ education length, is associated with subsequent developmental changes in the brains of children. Measurements of microstructural properties of the brain by DTI include mean diffusivity (MD) and FA. MD reflects the diffusivity of water molecules. It has been suggested that a wide range of tissue or cellular components (including capillaries, myelin, membrane, synapses, spines, axons, neuron, and glia) lower diffusivity in a non-specific manner^16^. FA reflects microstructural properties related to brain structural connectivity and myelination processes. Axon membrane thickness and diameter have been suggested to increase FA^16^. These measures can be affected by experiences that produce neural plasticity. In particular, MD is associated with cognitive intervention, daily habits, and genotypes associated with proteins involved in neural plasticity^17,18^.

In this study, analyses were performed on data gathered from Japanese children who had taken part in the longitudinal study. These children (age range: 5.7–18.4 years) were right-handed typically developing children without developmental disorders; they took part in the baseline experiment, underwent MRI scans, and took Wechsler IQ tests^19,20^. The children’s guardians answered questions regarding childhood SES, consisting of family income and parents’ education length. About a few years later, the majority of participants took part in the follow-up experiment, which involved further MRI scans and Wechsler IQ tests. MD and FA measures were collected using DTI together with regional gray matter volume (rGMV) using T1 weighted structural images processed with voxel-based morphometry (VBM).

First, cross-sectional analyses were performed using the data obtained from the baseline. The association of composite childhood SES score, which took account of family income and the average of the parents’ education duration) with IQ, rGMV, and FA of the white matter area, and MD of the gray and white matter areas after correction for age and sex were investigated through psychological analyses using the Wechsler IQ test, and whole-brain voxel-by-voxel multiple regression analyses. Then, longitudinal analyses were performed using data from both follow-up and baseline experiments. The associations of the composite childhood SES score with changes in outcome measures in the cross-sectional analyses after correction for age, sex, interval of the experiment, and the outcome value of the baseline experiment were investigated using multiple regression analyses. In longitudinal imaging analyses, we conducted a voxel-by-voxel whole-brain longitudinal multiple regression analysis that corrected the values of imaging measurements of the baseline scan at each voxel to increase sensitivity in order to detect an association between childhood SES and longitudinal neural changes^21^.

Based on previous work, it was hypothesized that childhood SES would be associated with subsequent changes in microstructural properties of the prefrontal cortex, medial temporal lobe, and left lateralized language-related regions. We basically hypothesized that childhood SES would be associated with greater increases in FA, and greater decreases in MD, as development during childhood and greater intelligence have both been found to be associated with higher or increased FA and lower or decreased MD^22,23^. However, as multiple studies, including those with large sample sizes, showed greater MD or increased MD was associated with greater competence at certain types of cognition and effects of cognitive training^24–26^, the opposite could not be ruled out. Given the associations of childhood SES with a wide range of cognitive mechanisms, paired with the unique characteristics of DTI, it is important to elucidate the longitudinal associations of childhood SES with microstructural properties of the brain for social and scientific advancements.

## Results

### Basic data

**Fig. 1:**
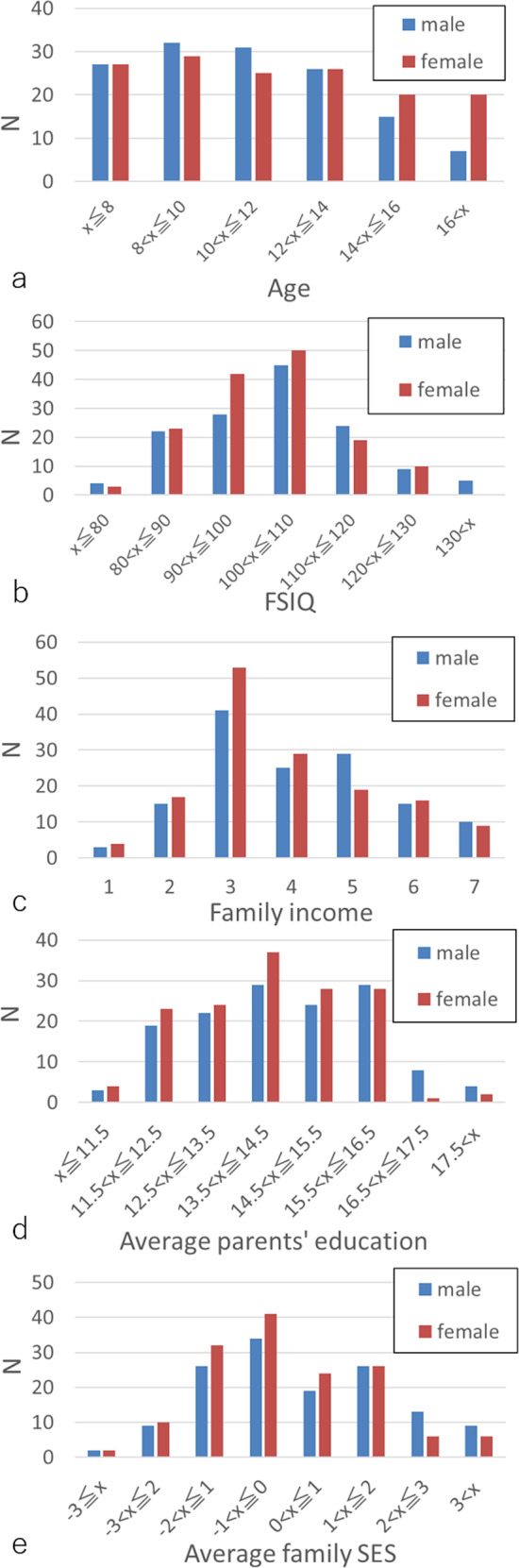
Distributions of the key variables of the study. Histograms showing (**a**) age, (b) FSIQ, (**c**) family income, (**d**) average parents’ education length, and (**e**) the composite childhood SES score of boys and girls in the sample.

The basic participant psychological data are provided in Table 1. The distribution of family income, average of parents’ education length, and the composite score for all participants are shown in Fig. 1.

**Table 1:** Psychological characteristics of the study participants (mean ± SD, range) in the cross-sectional (138 boys and 147 girls) and longitudinal imaging analyses (117 boys and 110 girls).

Measure	Boys	Girls
Age (years) (mean ± SD, range)	10.9 ± 2.8, 5.7–16.6 3.0 ± 0.3, 1.7–4.0	11.5 ± 3.3, 5.8–18.4 3.1 ± 0.3, 1.8–4.1
FSIQ (mean ± SD, range)	104.1 ± 12.9, 77–134 0.5 ± 9.0, −23–24	101.5 ± 11.1, 71–128 1.9 ± 9.3, −44–26
VIQ (mean ± SD, range)	105.4 ± 13.3, 72–152 −0.5 ± 10.3, −28–27	102.2 ± 13.0, 67–134 1.4 ± 10.2, −41–22
PIQ (mean ± SD, range)	101.7 ± 13.7, 62–136 1.6 ± 9.6, −23–26	100.0 ± 11.1, 73–129 2.3 ± 10.7, −50–32
Family annual income^a^	4.07 ± 1.49, 1–7	3.86 ± 1.47, 1–7
Average number of years of parents’ highest educational qualification	14.4 ± 1.7, 9–18.5	14.1 ± 1.5, 10.5–18.5
Z score of Family annual income + Z score of average number of years of parents’ highest educational qualification	0.19 ± 1.79, -4.5–4.08	−0.13 ± 1.58, −3.34–4.01

^a^Family annual income was classified as follows: 1, annual income below 2 million yen; 2, 2–4 million yen; 3, 4–6 million yen; 4, 6–8 million yen; 5, 8–10 million yen; 6, 10–12 million yen; 7, >12 million yen.

Data for the cross-sectional study are detailed in the upper lines for each metric, whereas the lower lines indicate data (the differences between two timepoints) for the longitudinal study. Metrics with only one line are for cross-sectional data.

### Cross-sectional behavioral analyses

**Fig. 2:**
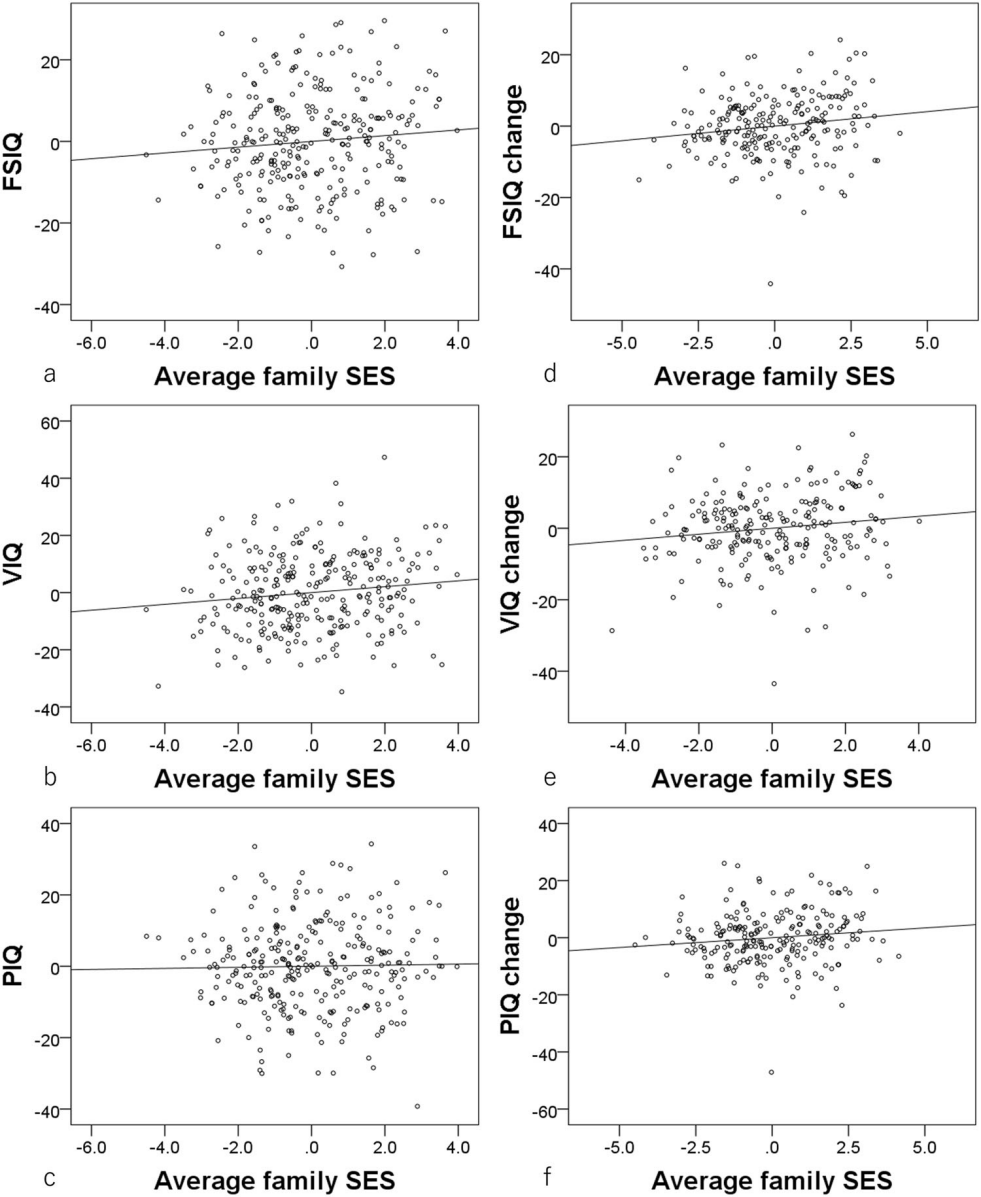
Associations between childhood SES and IQ measures, as well as changes across time. **a**–**c** Residual plots with trend lines depicting the correlations between residuals in multiple regression analyses with (**a**) FSIQ (significant, *N* = 284), (**b**) VIQ (significant, *N* = 284), and (**c**) PIQ (insignificant, *N* = 285) at baseline as a dependent variable, and childhood SES at baseline and other confounding factors as independent variables. **d**–**f** Residual plots with trend lines depicting the correlations between residuals in multiple regression analyses with baseline to follow-up experiment changes in (d) FSIQ (significant, *N* = 225), (**e**) VIQ (significant, *N* = 225), and (**f**) PIQ (insignificant, *N* = 225) as the dependent variables and childhood SES at baseline and other confounding factors as independent variables.

Multiple regression analyses at the baseline experiment timepoint revealed that, after correcting for confounding variables and multiple comparisons, higher SES was significantly associated with greater VIQ (*N* = 284, due to failure to obtain the proper VIQ data in one subject) and FSIQ (*N* = 284), but not PIQ (*N* = 285) (Fig. 2a, b, c, Table 2). Similar, weaker association patterns were observed when the contributions of family income and parents’ education length were analyzed separately (Table 3).

**Table 2:** Statistical values of associations between childhood SES and psychometric intelligence measures (cross-sectional multiple regression analyses) or their longitudinal changes (longitudinal multiple regression analyses).

Cross-sectional multiple regression analyses, Longitudinal multiple regression analyses, Dependent variables	*N*	Standardized beta (β) (95% CI)	*T*	*P* (uncorrected)	*P* (FDR)
FSIQ	284	0.099 (0–0.198)	1.646	0.050	0.042
VIQ	284	0.131 (0.033–0.230)	2.203	0.014	0.022
PIQ	285	0.020 (−0.079–0.120)	0.337	0.368	0.258
FSIQ change	225	0.151 (0.046–0.255)	2.375	0.009	0.022
VIQ change	225	0.137 (0.032–0.243)	2.149	0.016	0.022
PIQ change	225	0.114 (0.012–0.216)	1.854	0.033	0.035

Lower *p* values in FDR analyses compared with raw *p* values in two-stage sharpened methods occur when there are many strong signals (lower *p* values) among the analyses, which are proper^69^.

**Table 3:** Statistical values for the cross-sectional and longitudinal analyses of associations between family annual income or parents’ education length and psychometric intelligence measures.

Dependent variables	β (95% CI)	*t*	*P* (uncorrected)
*Cross-sectional multiple regression analysis (family annual income)*			
FSIQ	0.072 (−0.027–0.172)	1.196	0.116
VIQ	0.110 (0.011–0.210)	1.838	0.034
PIQ	−0.001<(−0.100–0.099)	−0.006	1.000
*Longitudinal multiple regression analysis (family annual income)*			
FSIQ change	0.121 (0.016–0.226)	1.909	0.029
VIQ change	0.092 (−0.013–0.198)	1.443	0.075
PIQ change	0.104 (0.002–0.206)	1.679	0.047
*Cross-sectional multiple regression analysis (parents’ education length)*			
FSIQ	0.093 (−0.005–0.191)	1.568	0.059
VIQ	0.110 (0.012–0.208)	1.861	0.032
PIQ	0.034 (−0.064–0.132)	0.568	0.570
*Longitudinal multiple regression analysis (parents’ education length)*			
FSIQ change	0.131 (0.027–0.236)	2.079	0.019
VIQ change	0.137 (0.032–0.241)	2.159	0.016
PIQ change	0.089 (−0.013–0.19)	1.447	0.075

### Longitudinal behavioral analyses

Multiple regression analyses revealed that, after correcting for confounding variables and multiple comparisons, higher baseline childhood SES was significantly associated with greater baseline to follow-up experiment increases in VIQ (*N* = 225), PIQ (*N* = 225), and FSIQ (*N* = 225) (Fig. 2d–f, Table 2). Similar, mostly weaker associations were observed when the contributions family income and parents’ education length were analyzed separately (Table 3).

### Cross-sectional neuroimaging analyses

**Fig. 3:**
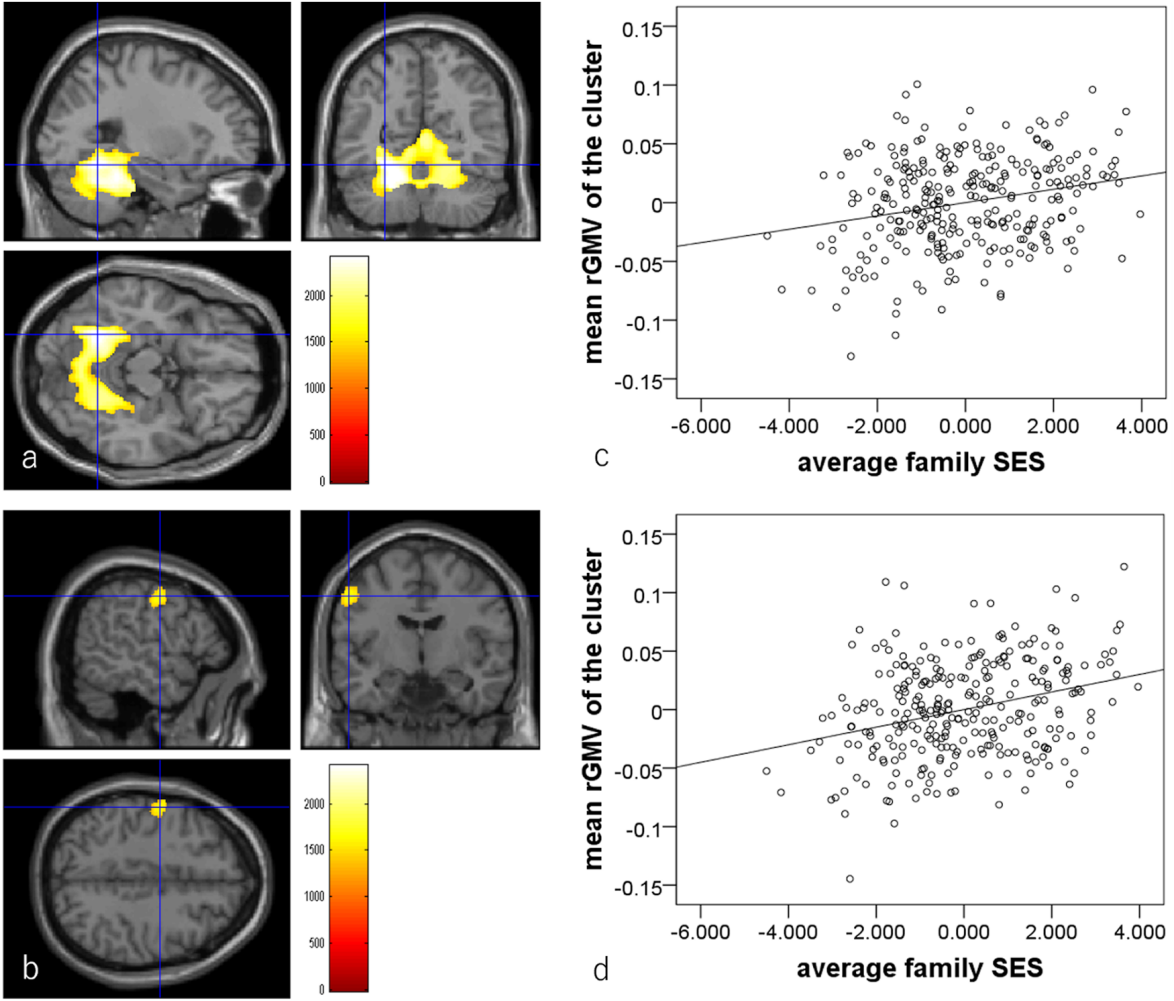
Associations between greater higher childhood SES and rGMV in cross-sectional analyses (*N* = 285). The results shown were obtained using a TFCE of *P* < 0.05 based on 5000 permutations. The results were corrected at the whole brain level. Significantly associated regions were overlaid on a “single subject” T1 image. The color represents the strength of the TFCE value. Positive associations between rGMV and childhood SES. Significant associations were observed in the anatomical cluster that primarily spread across the bilateral cerebellum, fusiform gyrus, lingual gyrus, precuneus, parahippocampal gyrus, and calcarine cortex (**a**) and in the anatomical cluster that primarily spread across the left pre- and post-central gyrus (**b**). **c**, **d** Partial residual plots with trend lines depicting associations between residuals in the multiple regression analyses. The mean rGMV from the significant clusters shown in (**a**) and (**b**) respectively were used as the dependent variables and childhood SES was the independent variable.

Multiple regression analyses using baseline experiment data revealed that, after correcting for confounding variables and multiple comparisons, higher childhood SES was significantly associated with greater rGMV in two anatomical clusters. The first anatomical cluster primarily spread across the bilateral cerebellum, fusiform gyrus, lingual gyrus, precuneus, parahippocampal gyrus, and calcarine cortex. The second anatomical cluster spread across the left pre- and post-central gyrus and left inferior parietal lobule (Fig. 3, Table 4, *N* = 285).

**Table 4:** Brain regions that exhibited significant positive associations between baseline childhood SES and rGMV.

Included gray matter areas^a^ (number of significant voxels in left and right side of each anatomical area)	*x*	*y*	*z*	TFCE value	Corrected *p* value (FWE)	Cluster size (voxel)	Cohen’s *r* with mean cluster values
Calcarine Cortex (R:21)/Fusiform gyrus (L:1523, R:1019)/Hippocampus (L:73, R:1)/Lingual gyrus (L:496, R:836)/Parahippocampal gyrus (L:307, R:138)/Precuneus (R:48)/Inferior temporal gyrus (L:1)/Cerebellum (L:5719, R:4736)/	−27	−55.5	−15	2407.48	0.008	18315	0.251
Inferior parietal lobule (L:4)/Post-central gyrus (L:424)/Precentral gyrus (L:44)/	−54	−7.5	43.5	1620.86	0.028	470	0.299

^a^Labelings of the anatomical regions of gray matter were mostly based on the WFU PickAtlas Tool (http://www.fmri.wfubmc.edu/cms/software#PickAtlas/) and on the PickAtlas automated anatomical labeling atlas option.

**Fig. 4:**
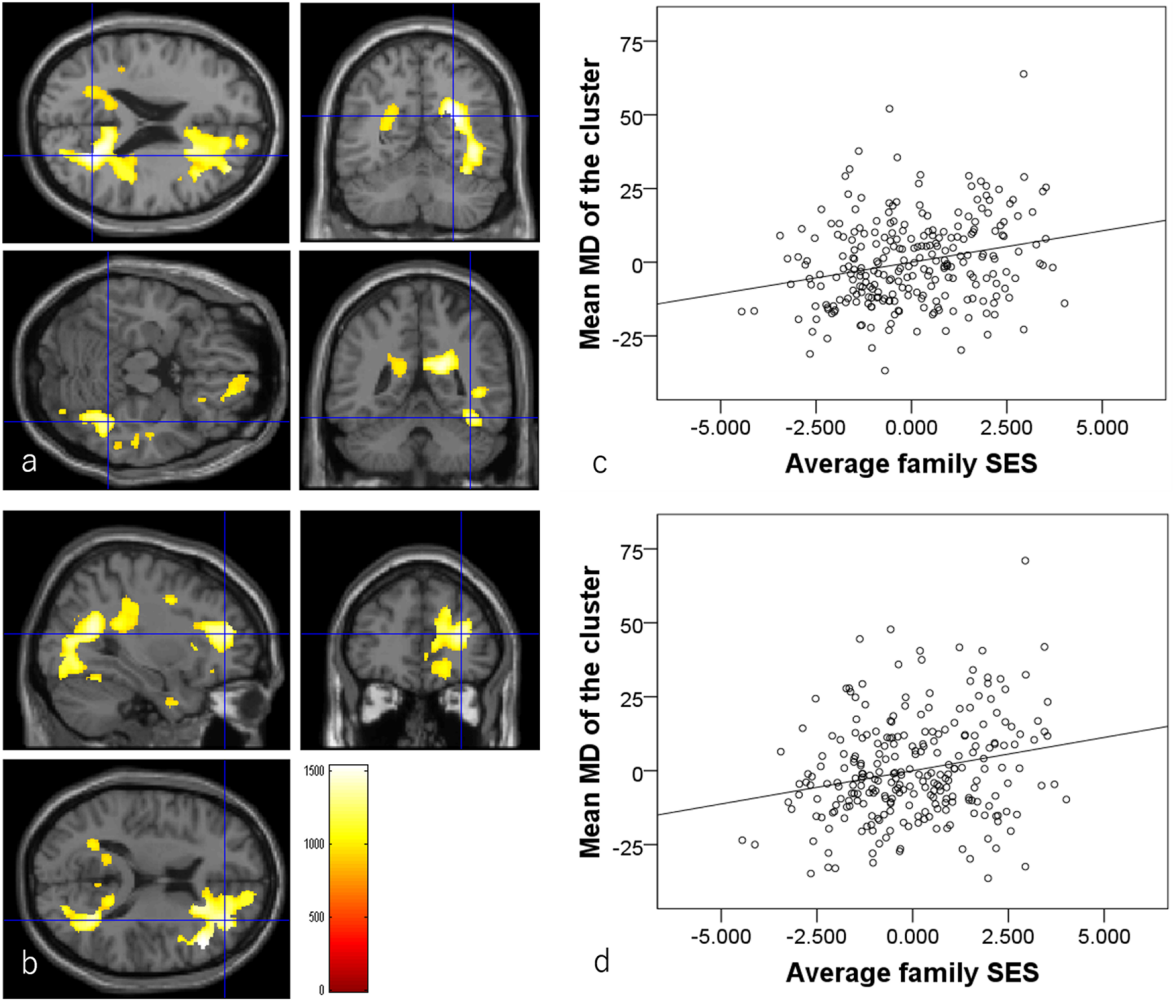
Associations between higher childhood SES and MD in cross-sectional analyses (*N* = 253). The results shown were obtained using a TFCE of *P* < 0.05 based on 5000 permutations. The results were corrected at the whole-brain level. Significantly associated regions were overlaid on a “single subject” T1 image. The color represents the strength of the TFCE value. Positive associations between MD and childhood SES. Significant associations were observed in the anatomical cluster that primarily spread across the right fusiform gyrus and surrounding areas (**a**) and in the anatomical cluster among the prefrontal cortex, striatum, anterior cingulate gray and white matter areas, and related regions (**b**). **c**, **d** Partial residual plots with trend lines depicting associations between residuals in the multiple regression analyses. The mean MD from the significant clusters shown in (**a**) and (**b**) respectively were used as the dependent variables and childhood SES was the independent variable.

Multiple regression analyses using baseline experiment data revealed that, after correcting for confounding variables and multiple comparisons, higher childhood SES was significantly associated with greater MD in multiple anatomical clusters that mostly spread across the right fusiform, lingual, and other occipital, temporal and medial posterior areas, cerebellum and right Rolandic operculum, the anatomical cluster that spread across left occipital and posterior medial areas, the anatomical cluster that spread among the prefrontal, striatum, and anterior cingulate gray and white matter areas, and so on (Fig. 4, Table 5, *N* = 253).

**Table 5:** Brain regions that exhibited significant positive correlation between childhood SES and MD.

Included gray matter areas^a^ (number of significant voxels in left and right side of each anatomical area)	Included large bundles^b^ (number of significant voxels in left and right side of each anatomical area)	*x*	*y*	*z*	TFCE value	Corrected *p* value (FWE)	Cluster size (voxel)	Cohen’s *r* with mean cluster values
Calcarine Cortex (R:366)/Middle cingulum (R:2)/Posterior cingulum (R:210)/Cuneus (R:177)/Fusiform gyrus (R:1493)/Heschl gyrus (R:38)/Insula (R:13)/Lingual gyrus (R:139)/Inferior occipital lobe (R:497)/Middle occipital lobe (R:274)/Superior occipital lobe (R:504)/Parahippocampal gyrus (R:37)/Post-central gyrus (R:55)/Precuneus (R:513)/Rolandic operculum (R:53)/Supramarginal gyrus (R:17)/Inferior temporal gyrus (R:1513)/Middle temporal gyrus (R:743)/Temporal pole (R:269)/Superior temporal gyrus (R:355)/Cerebellum (R:52)/	Body of corpus callosum (8)/Splenium of corpus callosum (572)/Retrolenticular part of internal capsule (R:14)/Superior corona radiata (R:13)/Posterior corona radiata (R:665)/Posterior thalamic radiation (R:414)/Sagittal stratum (R:15)/Cingulum (R:75)/Superior longitudinal fasciculus (R:496)/Tapatum (R:51)/	25.5	−58.5	22.5	1530.2	0.011	12328	0.186
Caudate (R:82)/Anterior cingulum (R:793)/Middle cingulum (R:39)/Inferior frontal operculum (R:128)/Inferior frontal orbital area (R:98)/Inferior frontal triangular (R:577)/Middle frontal medial area (R:506)/Middle frontal orbital area (R:34)/Middle frontal other areas (R:758)/Superior frontal medial area (R:459)/Superior frontal orbital area (R:418)/Superior frontal other areas (R:882)/Insula (R:48)/Putamen (R:21)/Rectus gyrus (R:147)/Rolandic operculum (R:29)/	Genu of corpus callosum (246)/Body of corpus callosum (175)/Anterior limb of internal capsule (R:37)/Anterior corona radiata (R:1206)/Superior corona radiata (R:193)/External capsule (R:12)/Cingulum (R:89)/Superior fronto-occipital fasciculus (R:6)/	49.5	24	13.5	1516.5	0.011	8972	0.178
Calcarine Cortex (L:8)/Posterior cingulum (L:33)/Cuneus (L:57)/Superior occipital lobe (L:26)/Precuneus (L:21)/	Splenium of corpus callosum (360)/Posterior corona radiata (L:57)/Posterior thalamic radiation (L:32)/Cingulum (L:37)/	−25.5	−58.5	16.5	1009.3	0.037	918	0.180
None	Body of corpus callosum (34)/Splenium of corpus callosum (26)/Posterior corona radiata (L:88)/	−18	−31.5	33	908.0	0.048	147	0.160
Angular gyrus (R:2)/Middle occipital lobe (R:43)/	None	40.5	−70.5	30	906.1	0.048	45	0.170
Rolandic operculum (L:3)/Supramarginal gyrus (L:2)/Superior temporal gyrus (L:1)/	Superior longitudinal fasciculus (L:36)/	−39	−37.5	25.5	902.6	0.049	83	0.157

^a^Labelings of the anatomical regions of gray matter were mostly based on the WFU PickAtlas Tool (http://www.fmri.wfubmc.edu/cms/software#PickAtlas/) and on the PickAtlas automated anatomical labeling atlas option.

^b^The anatomical labels and significant clusters of major white matter fibers were determined using the ICBM DTI-81 Atlas (http://www.loni.ucla.edu/).

Areas of significant positive correlation with MD and rGMV showed overlap in only 17 voxels distributed in the precuneus (7 voxels), right fusiform gyrus (7 voxels), and right cerebellum (3 voxels).

Multiple regression analyses using baseline experiment data revealed no significant associations between childhood SES and FA (N = 253).

**Fig. 5:**
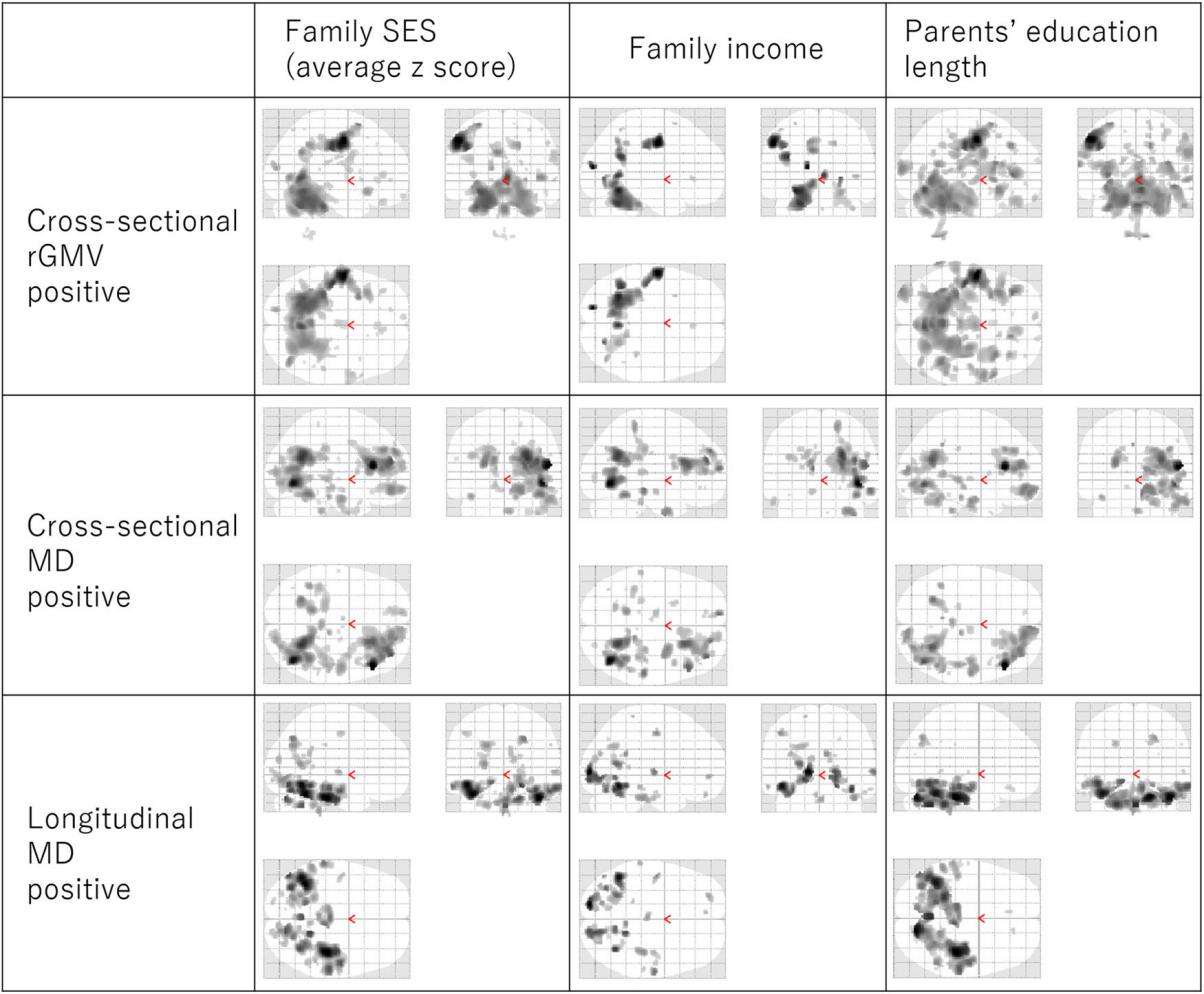
Associations between childhood SES measures and brain structures. The figures in the left, middle, and right columns represent the associations of average *z*-scores for family income and parents’ education length (left), family income alone (middle), and parents’ education length alone (right), respectively. The figures in the upper, middle, and lower lines represent positive childhood SES associations with rGMV in the cross-sectional analyses (upper, *N* = 285), MD in cross-sectional analyses (middle, *N* = 253), and MD in longitudinal analyses (lower, *N* = 200). The results shown were obtained using an uncorrected threshold of *P* < 0.005. Associated regions are superimposed on a glass brain image from SPM. Associations shared by family income, parents’ education length, and the average z-scores were observed.

Separate analyses for family income and parents’ educational length were conducted to observe their individual contributions to all significant neuroimaging findings (including longitudinal findings detailed in the following section). Although the two factors produced similar, results, parents’ education length showed overall stronger associations (Fig. 5).

### Longitudinal neuroimaging analyses

**Fig. 6:**
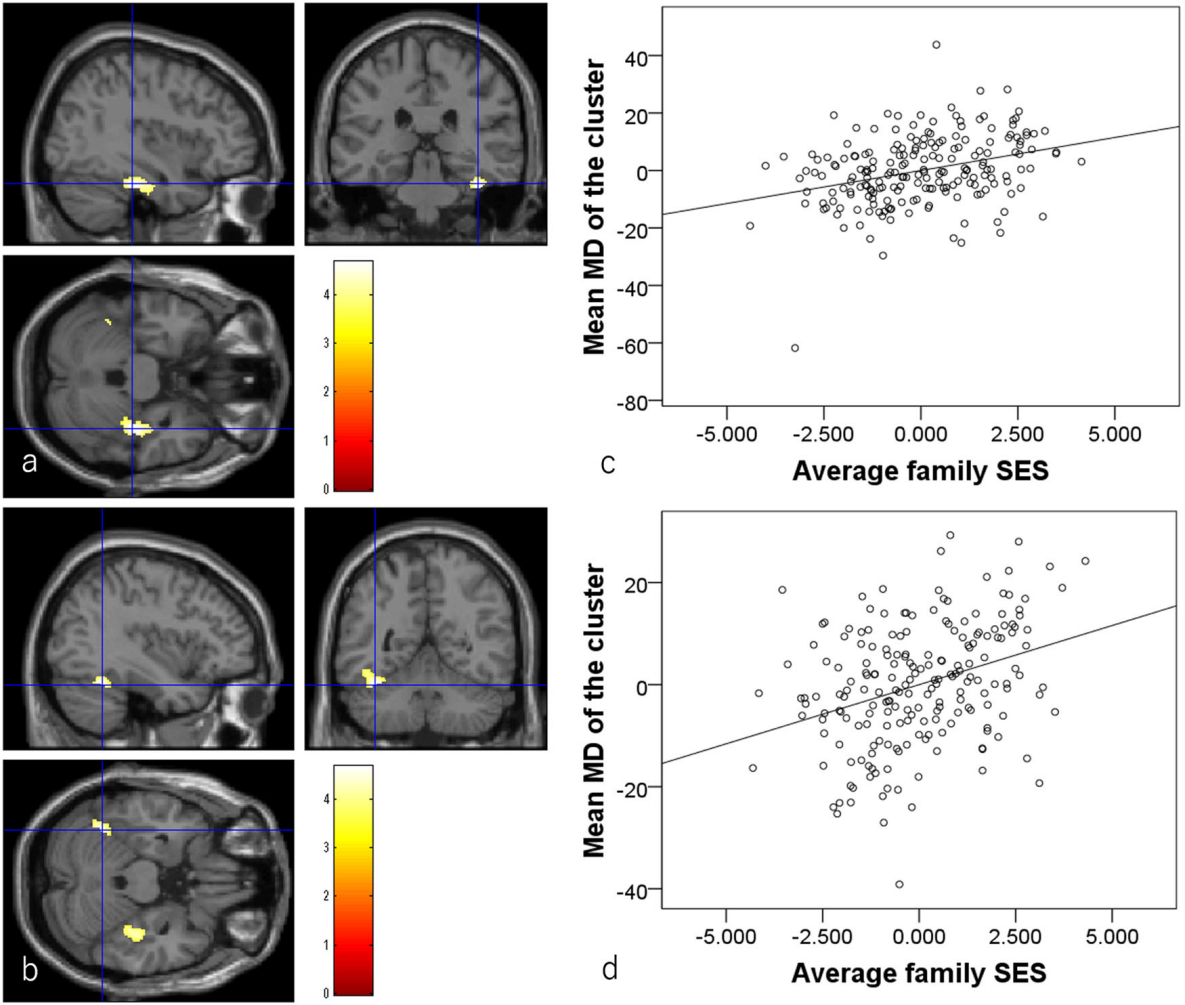
Associations between greater baseline to follow-up experimental MD changes and higher childhood SES in the longitudinal analysis (*N* = 200). **a**, **b** Areas of significant associations between greater MD change and higher childhood SES. Results are shown using a threshold of *P* < 0.05 corrected for multiple comparisons in cluster size tests, with a voxel-level cluster determining threshold of *P* < 0.05 (corrected for FDR). Results were corrected at the whole-brain level. Regions with significant correlations were overlaid on a “single participant” T1 image. The color represents the strength of the *T* value. Significant associations were observed in the anatomical cluster that primarily spread around the bilateral fusiform gyrus. **c**, **d** Partial residual plots with trend lines depicting associations between residuals in the multiple regression analyses. The mean MD from the significant clusters shown in (**a**) and (**b**) respectively were used as the dependent variables and childhood SES was the independent variable. Note as described in Methods, when the baseline value is adjusted in the multiple regression analyses, whether baseline to follow-up experimental value changes are used or follow-up experimental values are used as dependent variables, the *p* and *t* values will be the same. And since in this analysis, baseline to follow-up experimental time interval are adjusted, the displayed plots are not affected by the time interval.

Multiple regression analyses revealed that, after correcting for confounding variables and multiple comparisons, higher baseline childhood SES was significantly associated with greater baseline to follow-up experiment increases in MD in bilateral anatomical clusters that primarily spread throughout the bilateral fusiform gyrus and in the contingent cerebellum, parahippocampal gyrus, and inferior temporal areas (*N* = 200, Fig. 6, Table 6).

**Table 6:** Brain regions that exhibited significant positive associations between baseline childhood SES and baseline to follow-up experiment MD changes in longitudinal analyses.

Included gray matter areas^a^ (number of significant voxels in left and right side of each anatomical area)	Included large bundles^b^ (number of significant voxels in left and right side of each anatomical area)	*x*	*y*	*z*	*T* score	Corrected *p* value (FDR)***	Cluster size (voxel, corrected cluster level *p* value)***	Cohen’s *r* with mean cluster values
Fusiform gyrus (R:428)/Parahippocampal gyrus (R:12)/Inferior temporal gyrus (R:11)/Cerebellum (R:16)/	None	39	−30	−27	4.67	0.019	461 >0.001	0.221
Fusiform gyrus (L:355)/Inferior occipital lobe (L:40)/Inferior temporal gyrus (L:101)/Cerebellum (L:34)/	None	−37.5	−52.5	−24	4.64	0.019	520 >0.001	0.246

^a^Labelings of the anatomical regions of gray matter were mostly based on the WFU PickAtlas Tool (http://www.fmri.wfubmc.edu/cms/software#PickAtlas/) and on the PickAtlas automated anatomical labeling atlas option.

^b^The anatomical labels and significant clusters of major white matter fibers were determined using the ICBM DTI-81 Atlas (http://www.loni.ucla.edu/).

^c^Only the clusters that surpassed the extent threshold with the voxel-level cluster determining the threshold (*P* < 0.05, corrected for the false discovery rate) were noted.

Multiple regression analyses revealed no significant associations between baseline childhood SES and longitudinal rGMV (*N* = 226) or FA changes (*N* = 200). However, baseline to follow-up experiment rGMV trended toward an increase around the area of the right inferior parietal lobule [*P* = 0.005 when corrected at the cluster level (F.W.E) using the voxel-determining threshold of *p* < 0.001, but did not surpass the height threshold of *p* < 0.05 (FDR)].

### Post hoc correlation analyses between significant neuroimaging and psychological correlates of childhood SES

Next, we conducted partial correlation analyses between mean values of significant neuroimaging correlates of childhood SES and significant psychological correlates of family SES after controlling for confounding variables.

In cross-sectional analyses, among the abovementioned eight correlates of family SES (2 significant clusters in rGMV, and 6 significant clusters in MD) at baseline, after controlling for age and sex, only the mean MD values of the second largest cluster of MD analyses, which was spread around the right prefrontal cortex, showed a tendency toward positive correlation with baseline VIQ (*p* = 0.085, partial correlation coefficient = 0.087, one-tailed test). However, in cross-sectional analyses, childhood SES was specifically associated with VIQ at baseline, and the discrepancy between VIQ at baseline and PIQ at baseline showed stronger correlation with mean MD values of significant clusters and all significant clusters of MD; mean MD of all clusters but the smallest one showed significant positive correlation with the difference between VIQ at baseline and PIQ at baseline (*p* < 0.01, one-tailed tests).

In longitudinal analyses, among the two abovementioned imaging correlates of childhood SES (2 significant clusters in MD analyses) and three psychological correlates of family SES (changes in FSIQ, VIQ, PIQ), only the difference between mean MD value at follow-up and mean MD value at baseline of the significant cluster around the right fusiform gyrus showed a tendency of positive correlation with the difference between VIQ at follow-up and VIQ at baseline (*p* = 0.092, partial correlation coefficient = 0.095, one-tailed test).

Thus, unlike the associations between baseline childhood SES and longitudinal change in verbal IQ, longitudinal neuroimaging correlates of baseline childhood SES did not show a significant correlation with longitudinal change in verbal IQ. This may indicate that childhood SES affects verbal IQ and MD separately, or that MD is only indirectly associates with childhood SES, compared with physiological correlates such as tissue changes. It is also possible that childhood SES may be associated with VIQ through a wide range of neural mechanisms, and MD of particular areas is just one of these. However, these possibilities were not investigated in the present study.

### Supplemental analyses for interaction effects between age and childhood SES

To investigate whether there are interaction effects between age and childhood SES on psychological and anatomical measures (in other words, to test whether the associations between childhood SES and outcome variables vary with age), we performed supplemental psychological and whole-brain multiple regression analyses.

There were no significant interactions between age and childhood SES in all cross-sectional and longitudinal psychological and whole-brain analyses.

### Supplemental analyses of axial diffusivity and radial diffusivity

Though MD and FA were the primary outcome measures in this study, we also analyzed axial diffusivity (AD) and radial diffusivity (RD) to confirm whether these outputs might provide additional information.

**Fig. 7:**
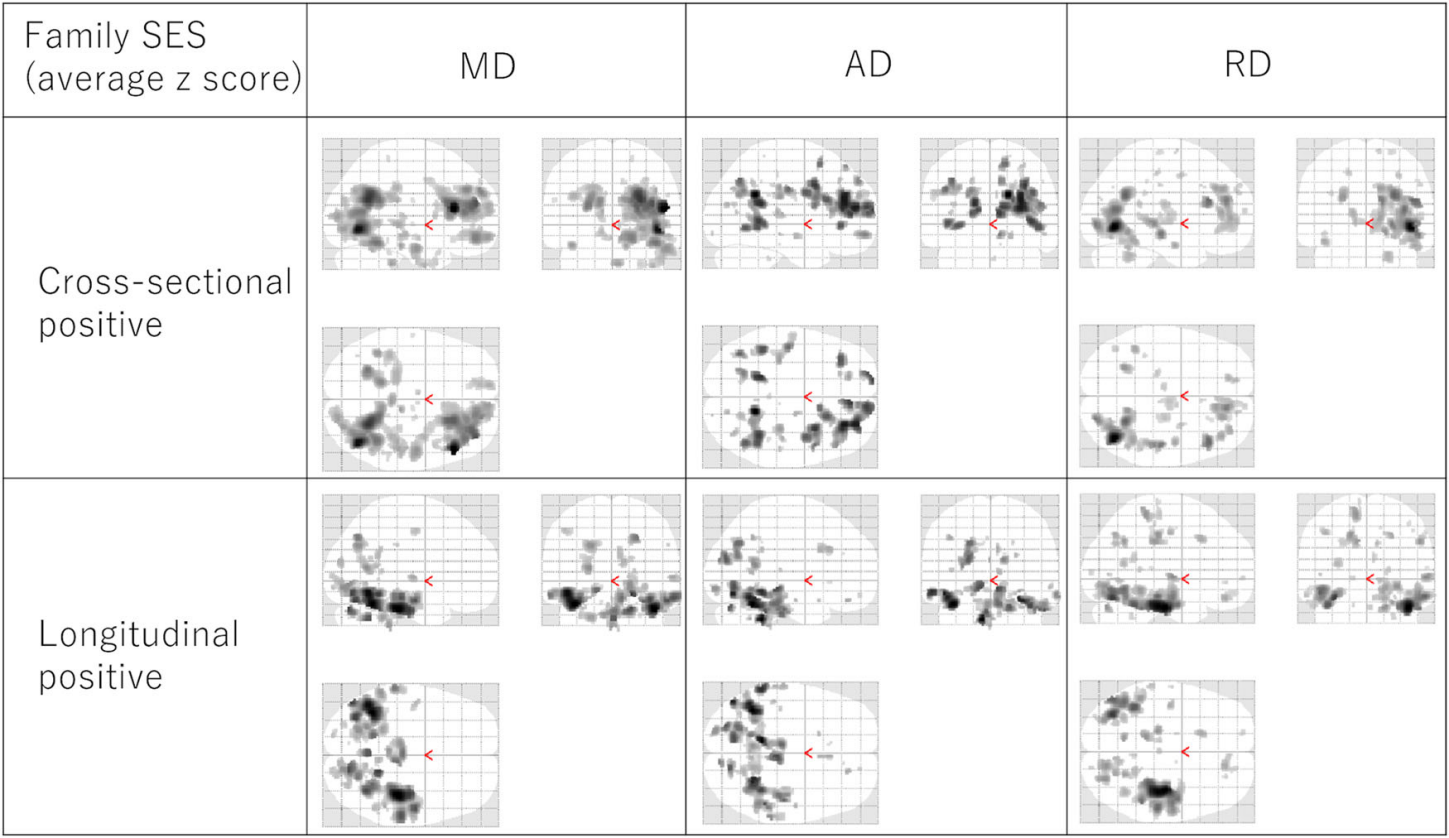
Associations between childhood SES and brain structures. The figures in the left, middle, and right columns represent the average z-scores for associations between family income and parents’ education with MD (left), AD (middle), and RD (right), respectively. The figures in the upper, middle, and lower rows represent positive childhood SES associations with diffusivity measurements in cross-sectional analyses (upper, *N* = 253) and longitudinal analyses (lower, *N* = 200). The results shown were obtained using an uncorrected threshold of *p* < 0.005. Associated regions are superimposed on a glass brain image from SPM. Similar associations were obtained by analysis of MD, AD, and RD.

Analyses of AD and RD showed similar results to those of MD, but weaker. Therefore, it was difficult to obtain meaningfully differential conclusions from these results (Fig. 7 presents the visual distribution of tendency of results of MD, AD, and RD).

### Supplemental analyses of multiple regression analyses with the length of videogame play as a covariate

We conducted additional multiple regression analyses that included the length of videogame play as an additional covariate in each analysis of the main analyses.

The sample sizes of the analyses in which the length of videogame play was added as an additional covariate were smaller than those of the main analyses (cross-sectional psychological and VBM analyses: *n* = 7, longitudinal and VBM psychological analyses: *n* = 6, cross-sectional DTI analyses: *n* = 6, and longitudinal DTI analyses *n* = 6).

**Fig. 8:**
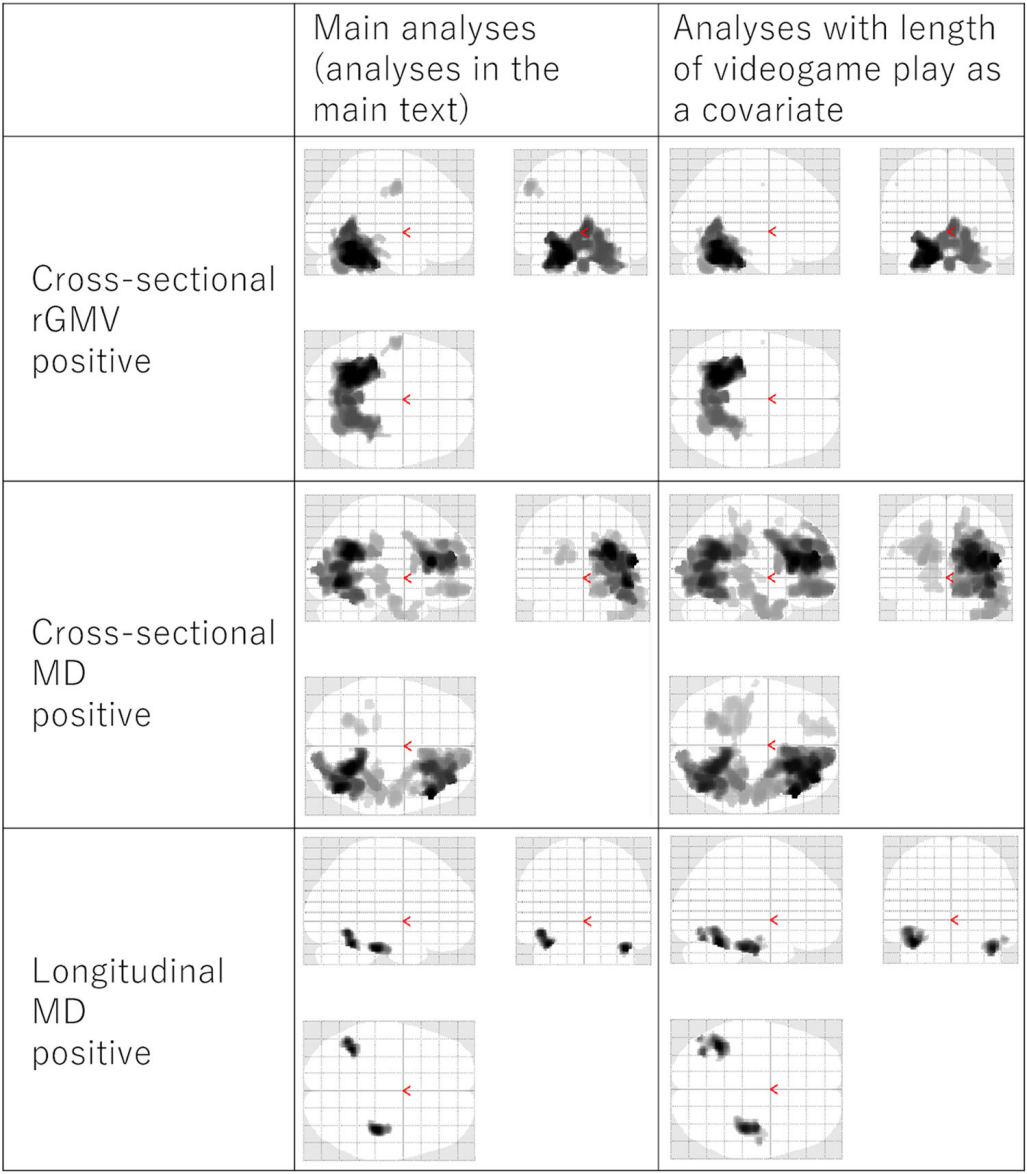
Comparisons of the results of imaging analyses in the main analyses and those that included length of videogame play as an additional covariate. The figures in the left and right columns represent the results of imaging analyses in the main analyses and those that included the length of videogame play as an additional covariate, respectively. The figures in the upper, middle, and lower rows represent positive childhood SES associations with rGMV in cross-sectional analyses (upper, left: *N* = 285, right: *N* = 278), MD changes in cross-sectional analyses (middle: *N* = 253, right: *N* = 247), and MD changes in longitudinal analyses (lower middle: *N* = 200, right: *N* = 194). The results shown were obtained using a TFCE of *p* < 0.05 based on 5,000 permutations in cross-sectional analyses (upper and middle rows). Results are shown using a threshold of *p* < 0.05 corrected for multiple comparisons in cluster size tests, with a voxel-level cluster-determining threshold of *p* < 0.05 (corrected for FDR) in longitudinal analyses (lower row). Associated regions are superimposed on a glass brain image from SPM. Similar associations were obtained by two types of analyses.

Overall, similar patterns of results were obtained between the two types of analyses. However, although all areas of the significant imaging results in the main analyses remained significant in these additional analyses, statistical values of some significant results in the main analyses became somewhat weak in these additional analyses and showed only statistical tendency (*p* = 0.102–0.107, corrected for FDR). (Table 7, Fig. 8).

**Table 7:** Comparisons of the results of psychological analyses in the main analyses and those that included length of videogame play as a covariate.

	Main analyses (multiple regression analyses of the main analyses)				Multiple regression analyses with the length of videogame play as a covariate				Mediation analyses that modeled length of videogame play as a mediating variable		
Dependent variables	β (95% CI)	*T*	*P* (unc)	*P* (FDR)	β	*T*	*P* (unc)	*P* (FDR)	*T*	*P* (unc)	*P* (FDR)
Cross-sectional FSIQ	0.099 (0–0.198)	1.646	0.050	0.042	0.082 (−0.018–0.183)	1.352	0.089	0.112	1.681	0.047	0.049
Cross-sectional VIQ	0.131 (0.033 –0.230)	2.203	0.014	0.022	0.111 (0.011–0.211)	1.832	0.034	0.086	2.122	0.017	0.033
Cross-sectional PIQ	0.020 (−0.079–0.120)	0.337	0.368	0.258	0.012 (−0.089–0.113)	0.195	0.423	0.444	0.492	0.311	0.272
LongitudinalFSIQ change	0.151 (0.046–0.255)	2.375	0.009	0.022	0.127 (0.020–0.235)	1.953	0.026	0.086	2.270	0.012	0.033
LongitudinalVIQ change	0.137 (0.032–0.243)	2.149	0.016	0.022	0.114 (0.006–0.221)	1.744	0.041	0.086	2.090	0.019	0.033
LongitudinalPIQ change	0.114 (0.012–0.216)	1.854	0.033	0.035	0.100 (−0.006–0.205)	1.562	0.060	0.095	1.764	0.040	0.049

### Supplemental mediation analyses with the length of videogame play as a mediator variable

We conducted one-tailed mediation analyses that modeled the length of videogame play as a mediator of the effects of childhood SES on IQ after confirming that the abovementioned additional multiple regression analyses including the length of videogame play as an additional covariate made some of the significant results in the main analyses marginally insignificant. This was done to investigate the possibility that childhood SES showed the hypothesized total positive effects (direct effect + indirect effect via videogame length) on IQ when the length of videogame play was considered as a mediator.

Mediation analyses revealed significant effects of childhood SES on FSIQ and VIQ in cross-sectional analyses and on changes of FSIQ, VIQ, and PIQ in longitudinal analyses after corrections for multiple comparisons (see Table 7 for statistical values). Among these, in cross-sectional analyses for FSIQ and VIQ and in longitudinal analyses for changes in FSIQ and VIQ, indirect effects of childhood SES via the length of videogame play on outcome IQ measures were confirmed at the level of *p* < 0.1, uncorrected for FDR.

## Discussion

The present study revealed cross-sectional and longitudinal associations of childhood SES with microstructural brain properties in children recruited from the Japanese general population. Partially consistent with our hypothesis, higher baseline childhood SES was associated with greater baseline experiment and baseline to follow-up experiment changes in MD in the bilateral fusiform gyri, which is a language-related brain region. Interestingly, this was despite the relationship between higher baseline childhood SES and greater baseline experiment and baseline to follow-up experiment changes in psychometric intelligence, which has previously been shown to be robustly associated with lower MD^23^. Finally, only in the cross-sectional analysis, there were significant associations between higher childhood SES and greater rGMV in the left pre- and post-central gyrus, areas spread across the fusiform, calcarine, lingual gyrus, hippocampus, and parahippocampal gyrus. These results are partly consistent with previous studies. These findings also advance the neuroscientific studies by demonstrating an association of childhood SES with brain microstructure, specifically that of the fusiform gyrus, and IQ. Even if there are some changes of characteristics between the subjects of the baseline experiment and those of the follow-up experiment, longitudinal analyses were conducted among subjects that participated in both of the experiments, so the difference cannot affect the findings. The wide age range was unlikely to confound the findings, as age was corrected for in the multiple regression analyses.

Reasons underlying the association of childhood SES with greater MD and IQ, which strengthens over time, remain unclear. The current results are counterintuitive, given that lower MD is generally accepted to be caused by greater hindrance of water diffusion resulting from increased variance in neural tissue components^27^. Further, as seen in our previous studies^22,23^, supplemental analyses showed that greater MD is robustly associated with greater age and greater psychometric intelligence in this sample. Therefore, in the present sample, greater IQ is associated with lower MD and greater childhood SES, but childhood SES is associated with greater MD. A previous study found that greater videogame play in this sample was associated with lower VIQ and greater MD in the initial cross-sectional analyses, and these associations strengthened over time^23^. Conversely, although lower MD is often associated with greater cognitive competence^18^, previous work has also shown that greater MD in brain regions corresponding to those related to social cognition is associated with increased social competence (e.g., empathizing, emotional intelligence)^24,25^. Further, working memory training improves untrained cognitive performance and increases MD in regions that are highly dopaminergic, including the lateral prefrontal cortex area that was positively associated with MD and childhood SES in the current study^26^. Other than these, it should be noted that there may be a wide range of potential mediating factors that influence and mediate the associations observed in the present study.

The mechanisms of the association between greater MD and cognitive competence also remain to be elucidated. In the present study, supplemental analyses of both RD and AD showed results similar to but statistically weaker than the results of MD analysis, suggesting that the observed associations are not due to directional changes such as alterations in axons or myeline^28^. It has previously been suggested that greater regional cerebral blood flow (which is supposed to increase MD) may mediate these associations^24^. Use-dependent and/or developmental synaptic pruning have also been suggested to be associated with adaptive neural changes related to cognitive competence, and these physiological changes may also increase MD^24^. These mechanisms may also influence the positive association between greater childhood SES and MD. Although the present rGMV analyses could not confirm changes in synaptic pruning, a previous cross-sectional study showed that greater childhood SES was associated with steeper age-related declines in cortical thickness of the fusiform gyrus among children^29^. It has been suggested that this may be due to the prolonged period of cortical thinning resulting from developmental synaptic pruning. Further, another longitudinal study demonstrated that childhood SES was associated with relative longitudinal rGMV reduction in the region surrounding the left middle temporal gyrus^10^. Notably, the current study found an association between greater childhood SES and greater rGMV in the cross-sectional analyses, which may contradict the synaptic pruning hypothesis.

Although the present study is a longitudinal observation study, it is difficult to predict the mechanisms by which childhood SES is associated with intellectual abilities, rGMV, and MD. However, we can suggest several possible mechanisms for the reference based on mechanisms suggested in previous studies (for review of the possible mechanisms and relevant supporting evidence, see ref. ^30^). First, prenatal or postnatal nutrition or reduced exposure to toxins in families with higher SES may lead to better development and neurocognitive differences. Second, greater childhood SES may lead to a better educational environment, with more cognitive stimulation, such as increased verbal and cognitive exposure, as well as increased likelihood to attend a cram school. These environments may lead to use-dependent neural plasticity and cognitive enhancement during development. Third, greater childhood SES leads to lower psychosocial stress and altered parental behaviors, which in turn leads to differential neurocognitive development. Fourth, greater childhood SES may be associated with genetic differences that correlate with greater IQ and relevant brain indices.

Significant MD findings in the fusiform gyrus in both models may be associated with this region’s role in reading and letter recognition^31^. The present findings are in line with the notion that childhood SES is associated with alterations in brain regions involved in language and literacy^5^. A previous functional imaging study in children with low childhood SES demonstrated a greater association between reading skills and functional activation of the fusiform gyrus^32^. Therefore, childhood SES may be associated with the microstructural properties of the fusiform gyrus through language and literacy.

The cross-sectional associations of greater childhood SES with greater psychometric intelligence and greater rGMV are mostly congruent with previous studies. This study showed cross-sectional associations of childhood SES with psychometric intelligence (full scale IQ and verbal IQ) as well as greater rGMV in the area around the left precentral and post-central gyrus as well as in the area spreading around the bilateral cerebellum, parahippocampal gyrus, hippocampus, lingual gyrus, right precuneus, and right calcarine cortex. These results are mostly in agreement with previous studies. Large sample studies have shown robust associations between greater childhood SES and increased psychometric intelligence in children e.g.,^33^. Cross-sectional studies have demonstrated associations between childhood SES and children’s literacy and language abilities for review, see^5^. Thus, the present cross-sectional IQ results were consistent with previous studies. Further, a number of cross-sectional neuroimaging studies have revealed associations between greater childhood SES and greater quantitative gray matter measures in areas related to language, the prefrontal cortex, and the medial temporal lobe^34^. Similar to the fusiform gyrus, the lingual gyrus is involved in word processing and reading^35^. The perisylvian areas are suggested to be involved in language and linguistic processes, and among them, the precentral gyrus is thought to be important for language output processes^35^. And the cerebellum is thought to be involved in articulatory function^36^. Although the hippocampus is known to play a role in memory processes, stress levels are thought to mediate its association with childhood SES for review, see^7^. Therefore, the present cross-sectional rGMV findings are consistent with previous studies. However, it should be noted that each area has multiple functions, and therefore, the associations of these regions could be via these other functions, which cannot be determined in the present study. For example, the precentral gyrus in the cerebellum is associated with motor functions in a wide range of movement, including writing. The cerebellum is also associated with various learning and associative processes^37,38^. Childhood SES may be associated with structures of these regions through these functions, or through the usage of these functions.

The results of longitudinal analyses concerning psychometric intelligence and rGMV require more scrutiny into previous studies. The direct associations between childhood SES and developmental intellectual changes have not been extensively investigated, and the associations may differ among samples. However, the present finding that increased childhood SES was associated with later IQ increases during the developmental state is consistent with previous work. For example, a previous cross-sectional study revealed children from higher/low SES backgrounds show a greater difference in psychometric intelligence at an older age compared to a younger age^39^. A longitudinal study in Costa Rica showed that, among children with chronic iron deficiency, children from lower SES backgrounds showed relative reductions in psychometric intelligence compared to children from higher SES backgrounds, and this gap widened with age.

In contrast to McDermott et al.^10^, the current study did not find any significant association between increased childhood SES and developmental decreases in rGMV. On the contrary, there was a trend toward an association between higher SES and greater developmental rGMV increases in the present study. The reasons for this discrepancy are not clear. It could be due to methodological differences (measuring cortical thickness compared to rGMV, corrections for baseline experiment measures, sample size) as well as cultural differences. Particularly, McDermott et al.^10^ used the Hollingshead two-factor index, in which a composite index was derived from the level of parental education and occupation, and when education and occupation were reported for both parents, the highest SES of two parents was used. Conversely, in the present study, we focused on family income as widely performed and the average education level of parents. Future studies should further investigate whether the differences in the effects of childhood SES arise from these methodological differences. Additionally, it is known that cultural differences and SES show interactive effects on parents’ involvement in education^40^, and such cultural differences between studies may affect findings as well.

This study has a few limitations. First, it is an observational longitudinal study, not an intervention study. Although childhood SES was associated with developmental cognitive and neuroanatomical changes, a causal effect of childhood SES on these measures cannot be concluded. Second, other family factors, such as industriousness or genetic factors, may affect parents’ childhood SES and children’s cognitive and neural development independently or in a synergistic manner. These issues may be effectively resolved through a well-designed intervention study or studies that control for genetic factors as covariates. Finally, this was a smaller study that lacked the power to investigate the interactive effects of sex and childhood SES, separate contributions of parents’ educational levels, and family income (poverty). This study was also unable to reveal which factors (e.g., education of children, stress, nutrition level) mediate the relationship between childhood SES and cognitive and structural-developmental changes. Future studies with more power may be able to address these questions. Furthermore, in this study, we recruited children of a wide age range (5–18 years old at baseline). Although this is common in many neuroimaging studies of the effects of childhood SES in children as summarized in the second paragraph of the Introduction, given gray matter structures show clear non-linear change as children develop^41^, this wide age range of samples may make it difficult to detect some of the associations between childhood SES and outcome variables. To supplement these points, we performed additional multiple regression analyses to investigate the interaction between age and childhood SES; no significant correlates of this interaction effect were found in any cross-sectional or longitudinal psychological and whole-brain analyses (see Results). Future studies may reveal this issue using data obtained from the experiment designed to detect these interaction effects.

In conclusion, higher childhood SES is directly or indirectly associated with greater psychometric intelligence, rGMV, and MD in the cross-sectional analyses. The longitudinal analyses indicated that the associations with psychometric intelligence and MD increased over development. This association was particularly pronounced in the fusiform gyrus, which is related to reading and letter recognition. Definitive conclusions regarding the causality of these relationships requires intervention and physiological studies. However, the current findings should be considered when developing and revising policies regarding education.

## Methods

### Participants

All participants were Japanese children recruited from the general population in the following manner (which is reproduced from our previous study^42^). All subjects were healthy Japanese children who were recruited in the following manner. First, we distributed 29,740 advertisements summarizing the study to various kindergartens, elementary schools, junior high schools, and high schools in Miyagi Prefecture, Japan. Then, 1423 parents of interested subjects contacted us by mail. Next, we mailed both a child version and a parent version of detailed study information to those parents. Then, 776 parents and subjects who were willing to participate contacted us again by mail. Subjects who had any history of malignant tumors, head trauma with a loss of consciousness lasting more than five minutes, developmental disorders, epilepsy, psychiatric diseases, claustrophobia, impaired color vision, routine visits to a hospital because of illness, congenital disorders, or routine medications (except daily drugs such as cold or anti-allergy medications) were excluded through a preliminary telephone interview, a mail-in health questionnaire, an oral interview and after participation.

The following descriptions have been largely reproduced from our previous study of the same project e.g.,^43^. In brief, we successfully collected brain magnetic resonance (MR) images from participants. We did not use specific diagnostic tools during the abovementioned recruitment and exclusion processes, though the second author is a radiologist and thoroughly checked the T1 weighted structural images for unfound neurological diseases before and after preprocessing of the image with VBM2 (http://dbm.neuro.uni-jena.de/wordpress/vbm/download/). And when the image quality was not good it was scanned again, when still the images of good quality were not obtained, the subjects were excluded from the analysis. We stipulated that only right-handed children could participate in the study in an advertisement used for subject recruitment and also confirmed that all subjects were right-handed using the self-report questionnaire, the “Edinburgh Handedness Inventory”^44^.

Following the Declaration of Helsinki^45^, written informed consent was obtained from each participant and his or her parent. Approval for these experiments was obtained from the Institutional Review Board of Tohoku University. Approximately three years following the baseline experiment (interval details are shown in Table 1), the follow-up experiment was conducted and included a majority of the participants from the baseline experiment.

From the data that were successfully obtained through the abovementioned procedure, ross-sectional imaging analyses were conducted using data from 285 participants (138 boys and 147 girls; mean age, 11.2 ± 3.1 years; range, 5.7–18.4 years). Longitudinal imaging analyses were conducted using data from 223 participants (115 boys and 108 girls; mean age, 14.2 ± 3.1 years; range, 8.4–21.7 years). The sample size of this study was comparable to the representative developmental brain structural study of the field before the initiation of this experiment and was expected to have enough statistical power^46^.

### Assessments of psychological variables

The following descriptions were largely reproduced from previous published work from the same project e.g.,^43,47^. In both the baseline and follow-up experiments, children’s Full Scale intelligence quotient (FSIQ) was measured using the Japanese version of the Wechsler Adult Intelligence Scale-Third Edition (WAIS-III) for participants aged 16 years or older^19^ or the Wechsler Intelligence Scale for Children-Third Edition (WISC-III) for participants younger than 16 years^20^. The tests were administered by trained examiners^19^. FSIQ, verbal IQ (VIQ), and performance IQ (PIQ) were calculated for each participant from their WAIS/WISC scores.

In the baseline experiment, the SES measure consisted of three questions answered by the participant’s guardian. The first concerned annual family income as previously reported e.g.,^43,47^. Annual income data were collected using discrete variables with the currency exchange rate set at $1 US (USD) = 100 yen: 1. annual income < $20,000 USD; 2. annual income $20,000–40,000 USD; 3. annual income $40,000–60,000 USD; 4. annual income $60,000–80,000 USD; 5. annual income $80,000–100,000 USD; 6. annual income $100,000–120,000 USD; 7. annual income ≥ $120,000 USD. The assigned 1–7 values were used in subsequent regression analyses. The remaining two questions concerned the highest educational qualification of each parent: (1) elementary school graduate or below; (2) junior high school graduate; (3) normal high school graduate; (4) graduate of a short term school completed after high school (such as a junior college); (5) university graduate; (6) master’s degree; and (7) doctorate. Each score was converted into the number of years taken to complete the degree following typical Japanese education system conventions: 1. 6 years; 2. 9 years; 3. 12 years; 4. 14 years; 5. 16 years; 6. 18 years; 7. 21 years. The average of the converted values of both parents was used in the analyses. This protocol followed the standard approach used by the Japanese government for evaluating SES. Although, we did not evaluate other factors such as job types, focusing on income and parent’s education level for evaluation of childhood SES is common to representative studies of the field e.g.,^11^. This study used the average z-scores for family annual income and average parental education length. The use of a composite score of multiple childhood SES measures is widely applied in the field and increases the sensitivity of the analyses^48^.

### Image acquisition

All images were collected using a 3-T Philips Intera Achieva scanner. No scanner version change has been performed during the experiment.

Three-dimensional, high-resolution, T1-weighted images were collected using a magnetization-prepared rapid gradient-echo sequence. The parameters were as follows: 240 × 240 matrix, repetition time = 6.5 ms, echo time = 3 ms, inversion time = 711 ms, field of view = 24 cm, and 162 slices at 1.0-mm slice thickness for a scan duration of 8 min and 3 s.

Diffusion-weighted data were collected using a spin-echo echo-planar imaging sequence (repetition time = 10 293 ms, echo time = 55 ms, *Δ* = 26.3 ms, *δ* = 12.2 ms, field of view = 22.4 cm, 2 × 2 × 2 mm^3^ voxels, 60 slices, SENSE reduction factor = 2, number of acquisitions = 1). The diffusion weighting was isotropically distributed along 32 directions (*b*-value = 1000 s/mm^2^). Additionally, a single image with no diffusion weighting (*b*-value = 0 s mm−2; *b*0 image) was acquired. The total scan time was 7 min 17 s. FA and MD maps were calculated from the collected images using a commercially available diffusion tensor analysis package on the MR consol. There are acquisitions for phase correction and for signal stabilization and these are not used as reconstructed images. MD and FA maps were calculated from the collected images using a commercially available diffusion tensor analysis package on the MR consol. This practice has been used in many of our previous studies^49–53^. Furthermore, the results of analyses using these image results were congruent with those of previous studies in which other methods were used^22,54^, suggesting the validity of this method. These procedures involved correction for motion and distortion caused by eddy currents. Calculations were performed according to a previously proposed method^55,56,57^.

Quality control of the images has been conducted by visual inspection.

The descriptions in this subsection were largely reproduced from our previous study of the same project using the same methods e.g.,^23,43^.

### Structural data preprocessing

Data preprocessing was performed using Statistical Parametric Mapping software (SPM12; Wellcome Department of Cognitive Neurology, London, UK) implemented in MATLAB (Mathworks Inc., Natick, MA, USA).

The following method descriptions have been largely reproduced from our previous study^58^. For the images used in whole-brain analyses, the new segmentation algorithm included in SPM12 was used to segment T1-weighted structural images from each individual at the baseline and follow-up experiment timepoints into 6 tissues. In this new segmentation process, default parameters were used, except that the Thorough Clean option was used to eliminate any odd voxel, affine regularization was performed with the International Consortium for Brain Mapping template for East Asian brains, and the sampling distance was set at 1 mm. We then proceeded to the diffeomorphic anatomical registration through exponentiated lie algebra (DARTEL) registration process implemented in SPM12. We used DARTEL import images of the two tissue probability maps from the abovementioned new segmentation process. First, the Dartel template was created using imaging data from all participants. Subsequently, the DARTEL procedures were performed for all the participants’ images. The resulting images were spatially normalized to the Montreal Neurological Institute space to give images with 1.5 × 1.5 × 1.5 mm^3^ voxels. In addition, we performed a volume change correction (modulation) by modulating each voxel with the Jacobian determinants derived from spatial normalization, which allowed us to determine regional differences in the absolute amount of brain tissue^59^. In VBM analyses, modulation procedures preserve the volume of the original images and transform it into the signal intensity in the normalized space^59^; modulated images are interpreted as image volume in VBM, in contrast to non-modulated images, which are often referred to as concentration or density images^56,57^. Subsequently, all images were smoothed by convolving them with an isotropic Gaussian kernel of 8 mm full width at half-maximum.

### Diffusion data preprocessing

Preprocessing was performed using SPM8 implemented in MATLAB. Briefly, baseline and follow-up experiment MD and FA images from participants were segmented and normalized with previously validated, two-step segmentation processes and modified DARTEL-based registration process method which utilized information from both of FA and MD maps, and the FA signal distribution within white matter areas for the normalization process^50^. The normalized MD images were masked using a custom mask image that is highly likely to be gray or white matter and smoothed by convolving them with an isotropic Gaussian kernel of 8 mm full width at half maximum. The normalized FA images were masked using a custom mask image that is highly likely to be white matter and smoothed by convolving them with an isotropic Gaussian kernel of 6 mm full width at half maximum. The descriptions in this subsection were mostly reproduced from our previous study using the same methods^23^.

This preprocessing procedure, generate findings congruent with those generated by tract-based spatial statistics in FA analyses^50^, achieved the accurate spatial normalization within white matter by using FA signal distribution for normalization, and solved the problem of partial volume effects of CSF’MD by applying the stringent mask, and allow analyses of MD within gray matter, effectively solving the problems of voxel-based DTI analyses and tract-based spatial statistics.

The full descriptions of this preprocessing procedure were provided below. The following method descriptions have been largely reproduced from our previous study^23,50^. First, each participant’s skull from the *b* = 0 image was stripped as previously described^52^; using the resulting image, diffusion images were linearly aligned to the skull-stripped *b* = 0 image template created previously^52^ to assist with the following procedures.

Subsequently, a previously validated two-step new segmentation algorithm of diffusion images and the previously validated DARTEL-based registration process^50^ which also utilized the FA signal distribution within white matter areas for normalization, all images, including gray matter segment [regional gray matter density (rGMD) map], white matter segment [regional white matter density (rWMD) map], cerebrospinal fluid (CSF) segments [regional CSF density (rCSFD) map] of diffusion images, were normalized. The voxel size of these normalized images was 1.5 × 1.5 × 1.5 mm^3^. In these processes, the template for the DARTEL process was created from the baseline experiment images of all subjects whose diffusion imaging data were obtained in the baseline experiment.

**Fig. 9:**
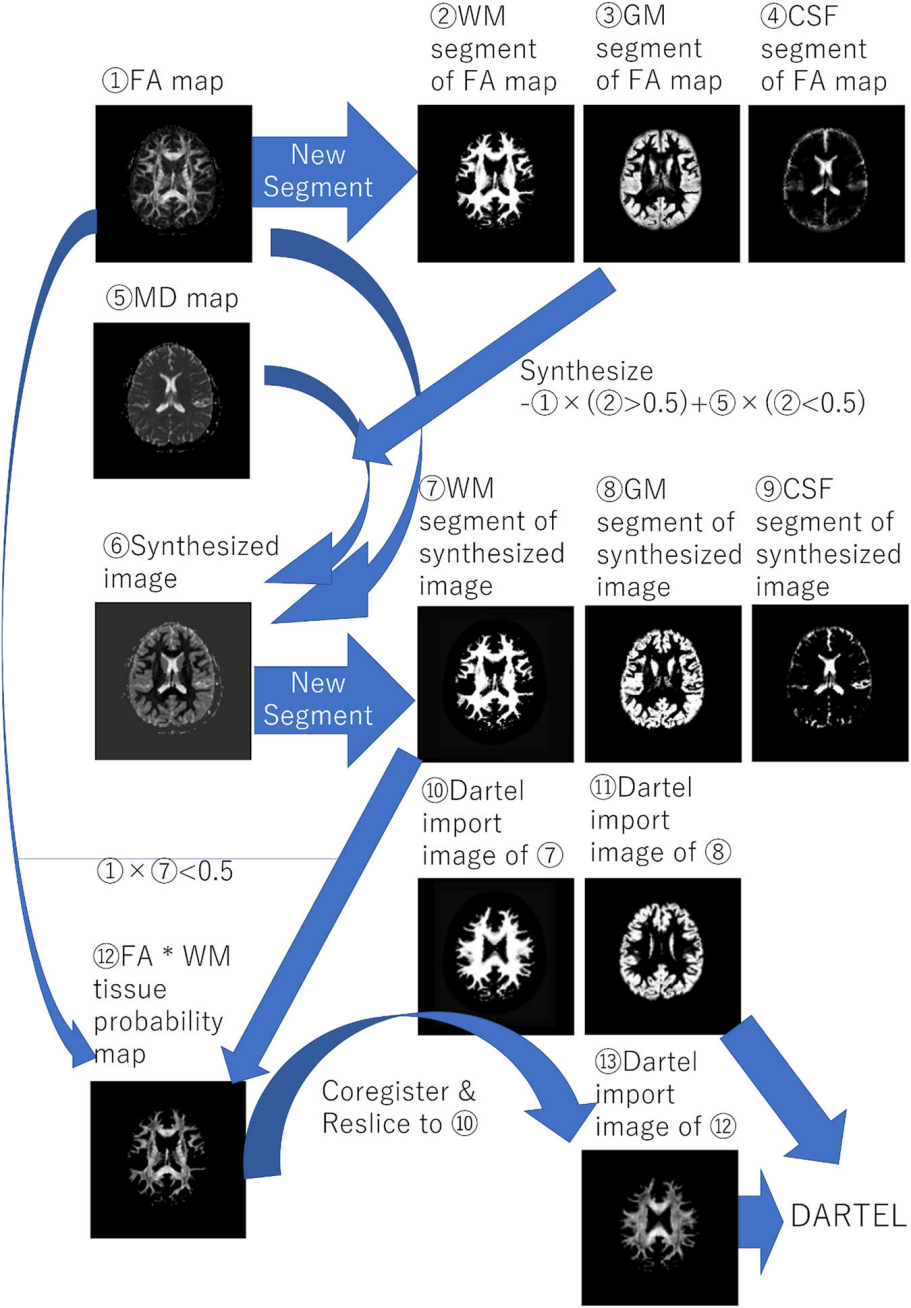
Diffusion images. A scheme of the preprocessing procedure of diffusion images.

The details of these procedures, which were also described in our previous study^50^, are as follows. Using the new segmentation algorithm implemented in SPM8, FA images (① in Fig. 9) of each individual were segmented into six tissues (first new segmentation) (②, ③, and ④ in Fig. 9 and other maps). The default parameters and tissue probability maps were used in this process, except that affine regularization was performed using the International Consortium for Brain Mapping template for East Asian brains and the sampling distance (approximate distance between sampled points when estimating the model parameters) was 2 mm. We then synthesized the FA image and MD map (① and ⑤ in Fig. 9). In the synthesized image (⑥ in Fig. 9), the area with a WM tissue probability >0.5 in the abovementioned new segmentation process was the FA image multiplied by −1 (hence, the synthesized image shows very clear contrast between WM and other tissues); the remaining area is the MD map (for details of this procedure, see below). The synthesized image from each individual was then segmented using the new segmentation algorithm implemented in SPM8 with the same parameters as above (second new segmentation), which generated ⑦, ⑧, ⑨, ⑩, and ⑪ in Fig. 9). This two-step segmentation process was adopted because the FA image has a relatively clear contrast between GM and WM, as well as between WM and CSF, and the first new segmentation step can segment WM from other tissues. On the other hand, MD map has clear contrast between GM and CSF and the second new segmentation can segment GM. Since the MD map alone lacks clear contrast between WM and GM, we must use a synthesized image (and the two-step segmentation process).

We then proceeded to the DARTEL registration process implemented in SPM8. We used the DARTEL import image of the GM tissue probability map produced in the second new segmentation process as the GM input for the DARTEL process. The WM input for the DARTEL process was created as follows. First, the raw FA image was multiplied by the WM tissue probability map from the second new segmentation process within the areas with a WM probability >0.5 (signals from other areas were set to 0 (⑫ in Fig. 9). Next, the FA image * WM tissue probability map was coregistered and resliced to the DARTEL import WM tissue probability image from the second segmentation (⑩ in Fig. 9), which created the DARTEL import image used (⑬ in Fig. 9). The DARTEL template was created using imaging data from the baseline experiment image of all subjects. Next, using the existing template, DARTEL procedures were performed for all images collected in this study. The parameters for these procedures were changed as follows to improve accuracy: The number of Gauss–Newton iterations performed within each outer iteration was set to 10 and, in each outer iteration, we used 8-fold more timepoints to solve the partial differential equations than the default values. The number of cycles used by the full multi-grid matrix solver was set to 8. The number of relaxation iterations performed in each multi-grid cycle was also set to 8. The resultant synthesized images were spatially normalized to Montreal Neurological Institute space. Using these parameters, the raw FA map, raw MD map, rGMD, rWMD and rCSFD map from the abovementioned second new segmentation process were normalized to give images with 1.5 × 1.5 × 1.5 mm^3^ voxels. The FA image * WM tissue probability map was used in the DARTEL procedures because it includes different signal intensities within WM tissues and the normalization procedure can take advantage of intensity differences to adjust the image to the template from the perspective of the outer edge of the tissue and within the WM tissue. No modulation was performed in the normalization procedure.

Next, average images for normalized rGMD and rWMD were created for all participants whose diffusion imaging data were obtained in the baseline experiment. Subsequently, for the analyses of MD images from the normalized images of the (a) MD, (b) rGMD, and (c) rCSFD maps, we created images where areas that were not strongly likely to be gray or white matter in our averaged normalized rGMD and rWMD images (defined by “gray matter tissue probability + white matter tissue probability < 0.99”) were removed (to exclude the strong effects of CSF on MD throughout analyses). These images were then smoothed (8 mm full-width half-maximum) and carried through to the second-level analyses of MD.

Next, we created average from the average image of normalized WM segmentation images of all subjects whose diffusion imaging data were obtained in the baseline experiment. And from the created mask image consisting of voxels with a WM signal intensity > 0.99. We then applied this mask image to the normalized FA image; therefore, we retained only areas that are highly likely to be white matter from the normalized FA images. These images were smoothed (6 mm full-width half-maximum) and carried through to the second-level analyses of FA. The lower smoothing values of FA maps, compared with those of rGMV and MD maps, were chosen as the contamination of signals from adjacent or intersecting tracts was particularly problematic in FA analyses.

### Statistics and reproducibility

#### Behavioral data analysis

Behavioral data were analyzed using Predictive Analysis Software, version 22.0.0 (SPSS Inc., Chicago, IL, USA; 2010). The following descriptions were largely reproduced from previous published work from the same project e.g.,^43^. One-tailed multiple regression analyses were used to investigate the hypothesized positive associations between (a) baseline childhood SES and VIQ, PIQ, and FSIQ in the baseline experiment phase and (b) baseline childhood SES and the baseline to follow-up experiment changes in VIQ, PIQ, and FSIQ. Sex, age (days after birth), and childhood SES score were included as independent variables in cross-sectional multiple regression analyses to separately analyze VIQ, PIQ, and FSIQ as dependent variables. Longitudinal multiple regression was used to separately analyze baseline to follow-up experiment changes in VIQ, PIQ, and FSIQ. In these analyses, the time interval between the baseline and follow-up experiment and the corresponding baseline IQ score were included as independent variables in addition to sex, age (days after birth), and childhood SES score. For example, when VIQ was the dependent variable, the baseline VIQ score was added as an independent variable in the model. In these analyses, results with a threshold of *P* < 0.05 were considered to be statistically significant after correcting for the false discovery rate (FDR) using the two-stage sharpened method^60^.

### Imaging data analysis

SPM8 was used for statistical analyses of imaging data. Cross-sectional whole brain multiple regression analysis was performed to investigate the association between childhood SES and brain images (rGMV, MD and FA). The same covariates described for the behavioral cross-sectional analyses were used.

Total intracranial volume was not included in the cross-sectional and longitudinal analyses because it is possible that higher childhood SES leads to enhanced head and body growth and widespread effects. When the effects of a variable are so widespread, the interpretation of results when the global effects are regressed out become difficult, not to mention most of the true differences were statistically insubstantial and will not be observed. Consistently, all previous studies of associations of childhood SES did not include total intracraniala volume as covariates^9–11^. However, even when total intracranial volume is corrected, in rGMV analyses, significant results of the Results section remain in the cerebellum, though the statistical strength became weaker and results of the left pre- and post-central gyrus became marginally insignificant.

We did not include various variables that may have been associated with childhood SES or neurocognitive outcome variables, such as reading habits, how much parents talk to children, or going to private school in multiple regression analyses. Previous studies did not control for any of these apparently relevant factors (for representative studies, see ref. ^9–11^). We believe this is probably because these variables are considered “mediating variables,” which do not necessarily have to be regressed out, in contrast to “confounding variables,” as childhood SES precedes all of these mediating variables. 1

Longitudinal whole-brain multiple regression analyses were used to analyze the associations between childhood SES and the follow-up experiment brain images (rGMV, MD, and FA). Age, sex, the baseline to follow-up experiment time interval, and corresponding baseline voxel-based brain imaging values were included as covariates. It was possible to correct for baseline imaging measurement effects on a voxel-by-voxel basis by using the biological parametric mapping tool (www.fmri.wfubmc.edu)^21^. Using this baseline experiment correction method, the *p* and t values are the same whether each dependent variable is the value of the follow-up experiment or the baseline to follow-up experiment change value for each measure. Therefore, these longitudinal analyses’ results using the follow-up experiment’s values as dependent variables were interpreted as the associations between childhood SES and baseline to follow-up brain changes.

Only voxels with an rGMV signal intensity of >0.10 were included for rGMV whole brain analyses. This intensity thresholding value is the default value stipulated in the manual of VBM created by the developer of VBM (http://dbm.neuro.uni-jena.de/vbm8/VBM8-Manual.pdf) and is a commonly used value. Though the value may suggest the voxel is more likely to be other tissues than gray matter, that would not matter as segmentation is performed. The MD and FA analyses were limited to their generated gray and white matter mask (MD) or white matter mask (FA), respectively (for the creation of the mask, see above).

For the cross-sectional whole-brain analyses, multiple comparison corrections were performed using threshold-free cluster enhancement (TFCE)^61^ with randomized (5,000 permutations) nonparametric testing using the TFCE toolbox (http://dbm.neuro.uni-jena.de/tfce/). We applied a threshold of family-wise error corrected at *P* < 0.05. Permutation tests allow exact control of error rates with minimal assumptions^62^. Among permutation tests, TFCE-based methods can take into account both amplitude and extent of effects without arbitrary selection of extent thresholds. Also, the software used to perform permutation tests requires reasonable computation time.

SPM8 was used here because of better compatibility of the software of TFCE and home-made script for analyses. As long as TFCE is used, the rationale for estimating the statistical significance is the same, and this would not matter to the results.

For the longitudinal analyses, multiple comparison corrections were performed using the FDR approach^63^. Areas that surpassed the extent threshold^64^ based on this cluster determining threshold were reported as has been performed in our previous study to control for false positives^23^. Different statistical thresholds were used because, although permutation tests can generally control false positive rates^65^, biological parametric mapping tool does not allow the use of TFCE and permutation in Windows. Therefore, the best available statistical method was chosen for each analysis. As are the cases of most of studies in the field, all p values of imaging analyses are those of one-tailed tests.

### Supplemental analyses of interaction effects between age and childhood SES

The dependent variables in the psychological and whole-brain analyses were the same as those in the cross-sectional and longitudinal analyses of the main analyses. In these supplemental analyses, statistical methods were also mostly the same as those of the main analyses, except that (a) age and average childhood SES are mean centered in all analyses, and (b) the variable of interaction of (mean centered) age and (mean centered) average childhood SES was added as an additional covariate.

### Supplemental analyses of axial diffusivity and radial diffusivity

For the generation of AD and RD images, first, images that were corrected for eddy current and motion as described above were forwarded to the calculation of these images. Calculation was performed using the artifact correction in diffusion MRI toolbox (http://www.diffusiontools.org), which is the extension software of Statistical Parametric Mapping (SPM) 12. Robust tensor fitting option which controls for whole brain volume outliers^66^, was used to calculate AD and RD. These images were then processed in the same way as MD images were processed and statistical analyses involving AD and RD were exactly performed in the same way as analyses of MD were performed. The results of MD, AD, and RD were compared using the lenient significance threshold (p < 0.005, uncorrected). The descriptions and methods of AD and RD processing are reproduced from our previous study^67^, with slight modifications.

### Supplemental analyses of multiple regression analyses with the length of videogame play as a covariate

We conducted additional multiple regression analyses that included the length of videogame play as an additional covariate in each analysis of the main analyses. In these additional analyses, the statistical designs and dependent and independent variables remained the same, excepting that the length of videogame play in the baseline experiment was added as an additional covariate. Information regarding the length of videogame play per weekday of each participant was gathered through the questionnaire. Findings related to this question were reported in our previous study^23^. These results were compared with those of the analyses in the main analyses.

### Supplemental mediation analyses that added the length of videogame play as a mediator variable

Mediation analyses can be performed by running a series of multiple regression analyses. If a relationship exists between an independent variable (A) and an outcome variable (B), a third variable might mediate the relationship between A and B if controlling for the variance attributable to the mediator variable reliably reduces the variance in B as explained by A.

Mediation analyses were conducted using an indirect macro designed for SPSS^68^. This macro uses bootstrapped sampling to estimate indirect mediation effects. Here, we tested how the length of videogame play mediated the relationship between childhood SES and IQ by testing six models for six outcome variables (FSIQ, VIQ, PIQ in cross-sectional analyses, changes in FSIQ, VIQ, and PIQ in longitudinal analyses). In this analysis, 5000 bootstrapped samples were drawn with replacement from the dataset to estimate a sampling distribution for the indirect mediation pathway (i.e., the pathway from childhood SES to the length of videogame play to each IQ measure).

In all six of the mediation analyses, the mediator variable was the length of videogame play at baseline, and the independent variable was baseline childhood SES. In three mediation analyses for cross-sectional analyses, FSIQ, VIQ, and PIQ at baseline were the dependent variables, respectively. In three mediation analyses for longitudinal analyses, the baseline to follow-up experiment changes in VIQ, PIQ, and FSIQ were the dependent variables. The mediation models for three cross-sectional analyses were controlled for variance in age and sex, and the mediation models for longitudinal analyses controlled for variance in age, sex, and the time interval between the baseline and follow-up experiment; baseline IQ scores corresponding to the dependent variable were included as independent variables.

Among these six mediation analyses, results with a threshold of *p* < 0.05 were considered to be statistically significant after correcting for the FDR using the two-stage sharpened method^60^.

### Reporting summary

Further information on research design is available in the Nature Research Reporting Summary linked to this article.

## Supplementary information

Reporting Summary

## Data Availability

All the experimental data obtained in the experiment of this study will be available to ones that were admitted in the ethics committee of Tohoku University, school of medicine. All the data sharing should be first admitted by the ethics committee of Tohoku University, school of medicine. The corresponding author is responsible for replying to this request and cooperating.

## Code availability

The names, versions, and parameters of the software that were used were provided in “Methods”.
