# Supplementary material for: Childhood socioeconomic status is associated with psychometric intelligence and microstructural brain development
Source: Commun Biol. 2021 Apr 29;4:470. doi: 10.1038/s42003-021-01974-w (PMC8084976; doi:10.1038/s42003-021-01974-w)
Supplement: Supplementary file 1 — Reporting Summary [file 42003_2021_1974_MOESM1_ESM.pdf]

nature research

Corresponding author(s): Hikaru Takeuchi

Last updated by author(s): 2021/01/25

Reporting Summary

Nature Research wishes to improve the reproducibility of the work that we publish. This form provides structure for consistency and transparency in reporting. For further information on Nature Research policies, see our Editorial Policies and the Editorial Policy Checklist.

Statistics

For all statistical analyses, confirm that the following items are present in the figure legend, table legend, main text, or Methods section.

n/a	Confirmed	
☐	☒	The exact sample size (*n*) for each experimental group/condition, given as a discrete number and unit of measurement
☐	☒	A statement on whether measurements were taken from distinct samples or whether the same sample was measured repeatedly
☐	☒	The statistical test(s) used AND whether they are one- or two-sided<br>*Only common tests should be described solely by name; describe more complex techniques in the Methods section.*
☐	☒	A description of all covariates tested
☐	☐	A description of any assumptions or corrections, such as tests of normality and adjustment for multiple comparisons
☐	☒	A full description of the statistical parameters including central tendency (e.g. means) or other basic estimates (e.g. regression coefficient) AND variation (e.g. standard deviation) or associated estimates of uncertainty (e.g. confidence intervals)
☐	☐	For null hypothesis testing, the test statistic (e.g. *F*, *t*, *r*) with confidence intervals, effect sizes, degrees of freedom and *P* value noted<br>*Give P values as exact values whenever suitable.*
☒	☐	For Bayesian analysis, information on the choice of priors and Markov chain Monte Carlo settings
☒	☐	For hierarchical and complex designs, identification of the appropriate level for tests and full reporting of outcomes
☐	☒	Estimates of effect sizes (e.g. Cohen's *d*, Pearson's *r*), indicating how they were calculated

Our web collection on statistics for biologists contains articles on many of the points above.

Software and code

Policy information about availability of computer code

Data collection	No software was used.
Data analysis	Behavioral data were analyzed using Predictive Analysis Software, version 22.0.0 (SPSS Inc., Chicago, IL, USA; 2010).<br>SPM8 was used for statistical analyses of cross-sectional imaging data.<br>SPM5 and it extension: biological parametric mapping tool (BPM; www.fmri.wfubmc.edu) was used for the statistical analyses of longitudinal imaging data.<br>For the cross-sectional whole brain analyses, multiple comparison corrections were performed using threshold-free cluster enhancement (TFCE) 52 with randomized (5,000 permutations) nonparametric testing using the TFCE toolbox (http://dbm.neuro.uni-jena.de/tfce/).

For manuscripts utilizing custom algorithms or software that are central to the research but not yet described in published literature, software must be made available to editors and reviewers. We strongly encourage code deposition in a community repository (e.g. GitHub). See the Nature Research guidelines for submitting code & software for further information.

Data

Policy information about availability of data

All manuscripts must include a data availability statement. This statement should provide the following information, where applicable:

- Accession codes, unique identifiers, or web links for publicly available datasets
- A list of figures that have associated raw data
- A description of any restrictions on data availability

All the experimental data obtained in the experiment of this study will be available to ones that were admitted in the ethics committee of Tohoku University, school of medicine. All the data sharing should be first admitted by the ethics committee of Tohoku University, school of medicine.

Field-specific reporting

Please select the one below that is the best fit for your research. If you are not sure, read the appropriate sections before making your selection.

☒ Life sciences ☐ Behavioural & social sciences ☐ Ecological, evolutionary & environmental sciences

For a reference copy of the document with all sections, see nature.com/documents/nr-reporting-summary-flat.pdf

Life sciences study design

All studies must disclose on these points even when the disclosure is negative.

Sample size

Cross-sectional imaging analyses were conducted using successfully obtained data from 285 participants (138 boys and 147 girls; mean age, 11.2 ± 3.1 years; range, 5.7–18.4 years). Longitudinal imaging analyses were conducted using successfully obtained data from 223 participants (115 boys and 108 girls; mean age, 14.2 ± 3.1 years; range, 8.4–21.7 years).

Data exclusions

All subjects were healthy Japanese children who were recruited in the following manner. First, we distributed 29,740 advertisements summarizing the study to various kindergartens, elementary schools, junior high schools, and high schools in Miyagi Prefecture, Japan. Then, 1,423 parents of interested subjects contacted us by mail. Next, we mailed both a child version and a parent version of detailed study information to those parents. Then, 776 parents and subjects who were willing to participate contacted us again by mail. Subjects who had any history of malignant tumors, head trauma with a loss of consciousness lasting more than five minutes, developmental disorders, epilepsy, psychiatric diseases, claustrophobia, impaired color vision, routine visits to a hospital because of illness, congenital disorders, or routine medications (except daily drugs such as cold or anti-allergy medications) were excluded through a preliminary telephone interview, a mail-in health questionnaire, an oral interview and after participation.
From the data that was successfully obtained through the abovementioned procedure, ross-sectional imaging analyses were conducted using data from 285 participants (138 boys and 147 girls; mean age, 11.2 ± 3.1 years; range, 5.7–18.4 years). Longitudinal imaging analyses were conducted using data from 223 participants (115 boys and 108 girls; mean age, 14.2 ± 3.1 years; range, 8.4–21.7 years).

Replication

There are no replication analyses.

Randomization

Randomization procedures are not irrelevant in this study.

Blinding

Blinding procedures are not irrelevant in this study.

Reporting for specific materials, systems and methods

We require information from authors about some types of materials, experimental systems and methods used in many studies. Here, indicate whether each material, system or method listed is relevant to your study. If you are not sure if a list item applies to your research, read the appropriate section before selecting a response.

Materials & experimental systems

n/a	Involved in the study
☒	☐ Antibodies
☒	☐ Eukaryotic cell lines
☒	☐ Palaeontology and archaeology
☒	☐ Animals and other organisms
☐	☒ Human research participants
☒	☐ Clinical data
☒	☐ Dual use research of concern

Methods

n/a	Involved in the study
☒	☐ ChIP-seq
☒	☐ Flow cytometry
☐	☒ MRI-based neuroimaging

Human research participants

Policy information about studies involving human research participants

Population characteristics

Participants. All participants were Japanese children recruited from the general population in the following manner (which is reproduced from our previous study 42). All subjects were healthy Japanese children who were recruited in the following manner. First, we distributed 29,740 advertisements summarizing the study to various kindergartens, elementary schools, junior high schools, and high schools in Miyagi Prefecture, Japan. Then, 1,423 parents of interested subjects contacted us by mail. Next, we mailed both a child version and a parent version of detailed study information to those parents. Then, 776 parents and subjects who were willing to participate contacted us again by mail. Subjects who had any history of malignant tumors, head trauma with a loss of consciousness lasting more than five minutes, developmental disorders, epilepsy, psychiatric diseases, claustrophobia, impaired color vision, routine visits to a hospital because of illness, congenital disorders, or routine medications (except daily drugs such as cold or anti-allergy medications) were excluded through a preliminary telephone interview, a mail-in health questionnaire, an oral interview and after participation.
The following descriptions have been largely reproduced from our previous study of the same project e.g., 43. In brief, , we

	successfully collected brain magnetic resonance (MR) images from participants. We did not use specific diagnostic tools during the abovementioned recruitment and exclusion processes, though the second author is a radiologist and thoroughly checked the T1 weighted structural images for unfound neurological diseases before and after preprocessing of the image with VBM2 (http://dbm.neuro.uni-jena.de/wordpress/vbm/download/). And when the image quality was not good it was scanned again, when still the images of good quality were not obtained, the subjects were excluded from the analysis. We stipulated that only right-handed children could participate in the study in an advertisement used for subject recruitment and also confirmed that all subjects were right-handed using the self-report questionnaire, the "Edinburgh Handedness Inventory" 44.
Recruitment	All subjects were healthy Japanese children who were recruited in the following manner. First, we distributed 29,740 advertisements summarizing the study to various kindergartens, elementary schools, junior high schools, and high schools in Miyagi Prefecture, Japan. Then, 1,423 parents of interested subjects contacted us by mail. Next, we mailed both a child version and a parent version of detailed study information to those parents. Then, 776 parents and subjects who were willing to participate contacted us again by mail. Subjects who had any history of malignant tumors, head trauma with a loss of consciousness lasting more than five minutes, developmental disorders, epilepsy, psychiatric diseases, claustrophobia, impaired color vision, routine visits to a hospital because of illness, congenital disorders, or routine medications (except daily drugs such as cold or anti-allergy medications) were excluded through a preliminary telephone interview, a mail-in health questionnaire, an oral interview and after participation.
Ethics oversight	Approval for these experiments was obtained from the Institutional Review Board of Tohoku University.

Note that full information on the approval of the study protocol must also be provided in the manuscript.

Magnetic resonance imaging

Experimental design

Design type	Longitudinal observation study.
Design specifications	In this study, analyses were performed on data gathered from Japanese children who had taken part in the longitudinal study. These children (age range: 5.7–18.4 years) were right-handed typically developing children without developmental disorders; they took part in the baseline experiment, underwent MRI scans, and took Wechsler IQ tests. The children's guardians answered questions regarding childhood SES, consisting of family income and parents' education length. About a few years later, the majority of participants took part in the follow-up experiment, which involved further MRI scans and Wechsler IQ tests. MD and FA measures were collected using DTI together with regional gray matter volume (rGMV) using T1 weighted structural images processed with voxel-based morphometry (VBM).
Behavioral performance measures	Assessments of psychological variables. The following descriptions were largely reproduced from previous published work from the same project e.g., 43, 47. In both the baseline and follow-up experiments, children's Full Scale intelligence quotient (FSIQ) was measured using the Japanese version of the Wechsler Adult Intelligence Scale-Third Edition (WAIS-III) for participants aged 16 years or older 19 or the Wechsler Intelligence Scale for Children-Third Edition (WISC-III) for participants younger than 16 years 20. The tests were administered by trained examiners 19. FSIQ, verbal IQ (VIQ), and performance IQ (PIQ) were calculated for each participant from their WAIS/WISC scores.<br>In the baseline experiment, the SES measure consisted of three questions answered by the participant's guardian. The first concerned annual family income as previously reported e.g., 43, 47. Annual income data were collected using discrete variables with the currency exchange rate set at $1 US (USD) = 100 yen: 1. annual income <$20,000 USD; 2. annual income $20,000–40,000 USD; 3. annual income $40,000–60,000 USD; 4. annual income $60,000–80,000 USD; 5. annual income $80,000–100,000 USD; 6. annual income $100,000–120,000 USD; 7. annual income ≥$120,000 USD. The assigned 1–7 values were used in subsequent regression analyses. The remaining two questions concerned the highest educational qualification of each parent: 1. elementary school graduate or below; 2. junior high school graduate; 3. normal high school graduate; 4. graduate of a short term school completed after high school (such as a junior college); 5. university graduate; 6. master's degree; and 7. doctorate. Each score was converted into the number of years taken to complete the degree following typical Japanese education system conventions: 1. 6 years; 2. 9 years; 3. 12 years; 4. 14 years; 5. 16 years; 6. 18 years; 7. 21 years. The average of the converted values of both parents was used in the analyses. This protocol followed the standard approach used by the Japanese government for evaluating SES. Although, we did not evaluate other factors such as job types, focusing on income and parent's education level for evaluation of childhood SES is common to representative studies of the field e.g., 11. This study used the average z-scores for family annual income and average parental education length. Use of a composite score of multiple childhood SES measures is widely applied in the field and increases the sensitivity of the analyses 48.<br><br>Mean, range, and SD were provided for each IQ measure in Table to confirm if subjects have performed the test properly.

Acquisition

Imaging type(s)	Three-dimensional, high-resolution, T1-weighted images, Diffusion-weighted data.
Field strength	3T
Sequence & imaging parameters	All images were collected using a 3-T Philips Intera Achieva scanner. No scanner version change has been performed during the experiment.<br>Three-dimensional, high-resolution, T1-weighted images were collected using a magnetization-prepared rapid gradient-echo sequence. The parameters were as follows: 240 × 240 matrix, TR = 6.5 ms, TE = 3 ms, TI = 711 ms, FOV = 24 cm,

and 162 slices at 1.0-mm slice thickness for a scan duration of 8 min and 3 s.
Diffusion-weighted data were collected using a spin-echo echo-planar imaging sequence (TR = 10293 ms, TE = 55 ms, Δ = 26.3 ms, δ = 12.2 ms, FOV = 22.4 cm, 2 × 2 × 2 mm3 voxels, 60 slices, SENSE reduction factor = 2, number of acquisitions = 1). The diffusion weighting was isotropically distributed along 32 directions (b-value = 1000 s/mm−2). Additionally, a single image with no diffusion weighting (b-value = 0 s mm−2; b0 image) was acquired. The total scan time was 7 min 17 s. FA and MD maps were calculated from the collected images using a commercially available diffusion tensor analysis package on the MR consol.

Area of acquisition: Whole-brain

Diffusion MRI: ☒ Used ☐ Not used

Preprocessing

Preprocessing software: SPM12 for T1 weighted structural images, and SPM8 for diffusion weighted image.

Normalization:

Structural data pre-processing. Data pre-processing was performed using Statistical Parametric Mapping software (SPM12; Wellcome Department of Cognitive Neurology, London, UK) implemented in MATLAB (Mathworks Inc., Natick, MA, USA). The following method descriptions have been largely reproduced from our previous study 56. For the images used in whole brain analyses, the new segmentation algorithm included in SPM12 was used to segment T1-weighted structural images from each individual at the baseline and follow-up experiment timepoints into 6 tissues. In this new segmentation process, default parameters were used, except that the Thorough Clean option was used to eliminate any odd voxel, affine regularization was performed with the International Consortium for Brain Mapping template for East Asian brains, and the sampling distance was set at 1 mm. We then proceeded to the diffeomorphic anatomical registration through exponentiated lie algebra (DARTEL) registration process implemented in SPM12. We used DARTEL import images of the 2 tissue probability maps from the abovementioned new segmentation process. First, the Dartel template was created using imaging data from all participants. Subsequently, the DARTEL procedures were performed for all the participants' images. The resulting images were spatially normalized to the Montreal Neurological Institute space to give images with 1.5 1.5 1.5 mm3 voxels. In addition, we performed a volume change correction (modulation) by modulating each voxel with the Jacobian determinants derived from spatial normalization, which allowed us to determine regional differences in the absolute amount of brain tissue (Ashburner and Friston 2000). Subsequently, all images were smoothed by convolving them with an isotropic Gaussian kernel of 8 mm full width at half maximum.

Diffusion data pre-processing. Pre-processing was performed using SPM8 implemented in MATLAB. Briefly, baseline and follow-up experiment MD and FA images from participants were segmented and normalized with previously validated, two-step segmentation processes and modified DARTEL-based registration process method which utilized information from both of FA and MD maps, and the FA signal distribution within white matter areas for the normalization process 50. The normalized MD images were masked using a custom mask image that is highly likely to be gray or white matter and smoothed by convolving them with an isotropic Gaussian kernel of 8 mm full width at half maximum. The normalized FA images were masked using a custom mask image that is highly likely to be white matter and smoothed by convolving them with an isotropic Gaussian kernel of 6 mm full width at half maximum. The descriptions in this subsection were mostly reproduced from our previous study using the same methods 23.
This preprocessing procedure, generate findings congruent with those generated by tract-based spatial statistics in FA analyses 50, achieved the accurate spatial normalization within white matter by using FA signal distribution for normalization, and solved the problem of partial volume effects of CSF'MD by applying the stringent mask, and allow analyses of MD within gray matter, effectively solving the problems of voxel based DTI analyses and tract-based spatial statistics.
The full descriptions of this preprocessing procedure were provided below. The following method descriptions have been largely reproduced from our previous study 23, 50. First, each participant's skull from the b = 0 image was stripped as previously described 52; using the resulting image, diffusion images were linearly aligned to the skull-stripped b = 0 image template created previously 52 to assist with the following procedures.
Subsequently, a previously validated two-step new segmentation algorithm of diffusion images and the previously validated DARTEL-based registration process 50 which also utilized the FA signal distribution within white matter areas for normalization, all images, including gray matter segment [regional gray matter density (rGMD) map], white matter segment [regional white matter density (rWMD) map], cerebrospinal fluid (CSF) segments [regional CSF density (rCSFD) map] of diffusion images, were normalized. The voxel size of these normalized images was 1.5 × 1.5 × 1.5 mm3. In these processes, the template for the DARTEL process was created from the baseline experiment images of all subjects whose diffusion imaging data were obtained in the baseline experiment.
The details of these procedures, which were also described in our previous study 50, are as follows. Using the new segmentation algorithm implemented in SPM8, FA images (① in Fig. 9) of each individual were segmented into six tissues (first new segmentation) (②, ③, and ④ in Fig. 9 and other maps). The default parameters and tissue probability maps were used in this process, except that affine regularization was performed using the International Consortium for Brain Mapping template for East Asian brains and the sampling distance (approximate distance between sampled points when estimating the model parameters) was 2 mm. We then synthesized the FA image and MD map (① and ⑤ in Fig. 9). In the synthesized image (⑥ in Fig. 9), the area with a WM tissue probability >0.5 in the abovementioned new segmentation process was the FA image multiplied by −1 (hence, the synthesized image shows very clear contrast between WM and other tissues); the remaining area is the MD map (for details of this procedure, see below). The synthesized image from each individual was then segmented using the new segmentation algorithm implemented in SPM8 with the same parameters as above (second new segmentation), which generated ⑦, ⑧, ⑨, ⑩, and ⑪ in Fig. 9). This two-step segmentation process was adopted because the FA image has a relatively clear contrast between GM and WM, as well as between WM and CSF, and the first new segmentation step can segment WM from other tissues. On the other hand, MD map has clear contrast between GM and CSF and the second new segmentation can segment GM. Since the MD map alone lacks clear contrast between WM and GM, we must use a synthesized image (and the two-step segmentation process).
We then proceeded to the DARTEL registration process implemented in SPM8. We used the DARTEL import image of the GM tissue probability map produced in the second new segmentation process as the GM input for the DARTEL process. The WM input for the DARTEL process was created as follows. First, the raw FA image was multiplied by the WM tissue probability

map from the second new segmentation process within the areas with a WM probability >0.5 (signals from other areas were set to 0 (⑫ in Fig. 9). Next, the FA image * WM tissue probability map was coregistered and resliced to the DARTEL import WM tissue probability image from the second segmentation (⑩ in Fig. 9), which created the DARTEL import image used (⑬ in Fig. 9). The DARTEL template was created using imaging data from the baseline experiment image of all subjects. Next, using the existing template, DARTEL procedures were performed for all images collected in this study. The parameters for these procedures were changed as follows to improve accuracy: The number of Gauss–Newton iterations performed within each outer iteration was set to 10 and, in each outer iteration, we used 8-fold more timepoints to solve the partial differential equations than the default values. The number of cycles used by the full multi-grid matrix solver was set to 8. The number of relaxation iterations performed in each multi-grid cycle was also set to 8. The resultant synthesized images were spatially normalized to Montreal Neurological Institute space. Using these parameters, the raw FA map, raw MD map, rGMD, rWMD and rCSFD map from the abovementioned second new segmentation process were normalized to give images with 1.5 × 1.5 × 1.5 mm3 voxels. The FA image * WM tissue probability map was used in the DARTEL procedures because it includes different signal intensities within WM tissues and the normalization procedure can take advantage of intensity differences to adjust the image to the template from the perspective of the outer edge of the tissue and within the WM tissue. No modulation was performed in the normalization procedure.
Next, average images for normalized rGMD and rWMD were created for all participants whose diffusion imaging data were obtained in the baseline experiment. Subsequently, for the analyses of MD images from the normalized images of the (a) MD, (b) rGMD, and (c) rCSFD maps, we created images where areas that were not strongly likely to be gray or white matter in our averaged normalized rGMD and rWMD images (defined by "gray matter tissue probability + white matter tissue probability < 0.99") were removed (to exclude the strong effects of CSF on MD throughout analyses). These images were then smoothed (8 mm full-width half-maximum) and carried through to the second-level analyses of MD.
Next, we created average from the average image of normalized WM segmentation images of all subjects whose diffusion imaging data were obtained in the baseline experiment. And from the created mask image consisting of voxels with a WM signal intensity > 0.99. We then applied this mask image to the normalized FA image; therefore, we retained only areas that are highly likely to be white matter from the normalized FA images. These images were smoothed (6 mm full-width half-maximum) and carried through to the second-level analyses of FA. The lower smoothing values of FA maps, compared with those of rGMV and MD maps, were chosen as the contamination of signals from adjacent or intersecting tracts was particularly problematic in FA analyses.

Normalization template

Described in "Normalization space". The standardized space is MNI305.

Noise and artifact removal

There are acquisitions for phase correction and for signal stabilization and these are not used as reconstructed images. MD and FA maps were calculated from the collected images using a commercially available diffusion tensor analysis package on the MR consol. This practice has been used in many of our previous studies 49, 50, 51, 52, 53. Furthermore, the results of analyses using these image results were congruent with those of previous studies in which other methods were used 22, 54, suggesting the validity of this method. These procedures involved correction for motion and distortion caused by eddy currents. Calculations were performed according to a previously proposed method 55.

Volume censoring

Quality control of the images has been conducted by visual inspection.

Statistical modeling & inference

Model type and settings

SPM8 was used for statistical analyses of imaging data. Cross-sectional whole brain multiple regression analysis was performed to investigate the association between childhood SES and brain images (rGMV, MD, and FA). The same covariates described for the behavioral cross-sectional analyses were used.
Total intracranial volume was not included in the cross-sectional and longitudinal analyses because it is possible that higher childhood SES leads to enhanced head and body growth and widespread effects. When the effects of a variable are so widespread, the interpretation of results when the global effects are regressed out become difficult58, not to mention most of the true differences were statistically insubstantial and will not be observed. Consistently, all previous studies of associations of childhood SES did not include total intracraniala volume as covariates9, 10, 11. However, even when total intracranial volume is corrected, in rGMV analyses, significant results of the Results section remain in the cerebellum, though the statistical strength became weaker and results of the left pre- and post- central gyrus became marginally insignificant.
We did not include various variables that may have been associated with childhood SES or neurocognitive outcome variables, such as reading habits, how much parents talk to children, or going to private school in multiple regression analyses. Previous studies did not control for any of these apparently relevant factors (for representative studies, see ref9, 10, 11). We believe this is probably because these variables are considered "mediating variables," which do not necessarily have to be regressed out, in contrast to "confounding variables," as childhood SES precedes all of these mediating variables. 1
Longitudinal whole brain multiple regression analyses were used to analyze the associations between childhood SES and the follow-up experiment brain images (rGMV, MD, and FA). Age, sex, the baseline to follow-up experiment time interval, and corresponding baseline voxel-based brain imaging values were included as covariates. It was possible to correct for baseline imaging measurement effects on a voxel-by-voxel basis by using the biological parametric mapping tool (www.fmri.wfubmc.edu)21. Using this baseline experiment correction method, the p and t values are the same whether each dependent variable is the value of the follow-up experiment or the baseline to follow-up experiment change value for each measure. Therefore, these longitudinal analyses' results using the follow-up experiment's values as dependent variables were interpreted as the associations between childhood SES and baseline to follow-up brain changes.
Only voxels with an rGMV signal intensity of >0.10 were included for rGMV whole brain analyses. This intensity thresholding value is the default value stipulated in the manual of VBM created by the developer of VBM (http://dbm.neuro.uni-jena.de/vbm8/VBM8-Manual.pdf) and is a commonly used value. Though the value may suggest the voxel is more likely to be other tissues than gray matter, that would not matter as segmentation is performed. The MD and FA analyses were limited to their generated gray and white matter mask (MD) or white matter mask (FA), respectively (for the creation of the mask, see above).

Effect(s) tested	Effects of the average z-scores for family annual income and average parental education length.

Specify type of analysis: ☒ Whole brain ☐ ROI-based ☐ Both

Statistic type for inference (See Eklund et al. 2016)	For the cross-sectional whole brain analyses, multiple comparison corrections were performed using threshold-free cluster enhancement (TFCE) 59 with randomized (5,000 permutations) nonparametric testing using the TFCE toolbox (http://dbm.neuro.uni-jena.de/tfce/). We applied a threshold of family-wise error corrected at P < 0.05. Permutation tests allow exact control of error rates with minimal assumptions60. Among permutation tests, TFCE-based methods can take into account both amplitude and extent of effects without arbitrary selection of extent thresholds. Also, the software used to perform permutation tests requires reasonable computation time.<br><br>For the longitudinal analyses, multiple comparison corrections were performed using the false discovery rate (FDR) approach 61. Areas that surpassed the extent threshold 62 based on this cluster determining threshold were reported as has been performed in our previous study to control for false positives 23. Different statistical thresholds were used because, although permutation tests can generally control false positive rates 63, biological parametric mapping tool does not allow the use of TFCE and permutation in Windows. Therefore, the best available statistical method was chosen for each analysis. As are the cases of most of studies in the field, all p values of imaging analyses are those of one-tailed tests.
Correction	Described in Statistic type for inference.

Models & analysis

n/a	Involved in the study
☒	☐ Functional and/or effective connectivity
☒	☐ Graph analysis
☒	☐ Multivariate modeling or predictive analysis
